# Photoresponsive Porphyrin Nanotubes of *Meso*-tetra(4-Sulfonatophenyl)Porphyrin and Sn(IV) *meso*-tetra(4-pyridyl)porphyrin

**DOI:** 10.3389/fchem.2019.00351

**Published:** 2019-05-16

**Authors:** Ekaterina A. Koposova, Andreas Offenhäusser, Yuri E. Ermolenko, Yulia G. Mourzina

**Affiliations:** ^1^Forschungszentrum Jülich, Institute of Complex Systems-8 (Bioelectronics), Jülich, Germany; ^2^Institute of Chemistry, Saint-Petersburg State University, Saint-Petersburg, Russia

**Keywords:** porphyrin nanotubes, Sn(IV) porphyrin, *meso*-tetra(4-sulfonatophenyl)porphyrin, π-tecton, supramolecular nanoassembly, photoconductivity, chemiresistor, salicylate

## Abstract

Porphyrin macrocycles and their supramolecular nanoassemblies are being widely explored in energy harvesting, sensor development, catalysis, and medicine because of a good tunability of their light-induced charge separation and electron/energy transfer properties. In the present work, we prepared and studied photoresponsive porphyrin nanotubes formed by the self-assembly of *meso*-tetrakis(4-sulfonatophenyl)porphyrin and Sn(IV) *meso*-tetra(4-pyridyl)porphyrin. Scanning electron microscopy and transmission electron microscopy showed that these tubular nanostructures were hollow with open ends and their length was 0.4–0.8 μm, the inner diameter was 7–15 nm, and the outer diameter was 30–70 nm. Porphyrin tectons, H_4_${\text{TPPS}}_{4}^{2-}$ : Sn(IV)TPyP^4+^, self-assemble into the nanotubes in a ratio of 2:1, respectively, as determined by the elemental analysis. The photoconductivity of the porphyrin nanotubes was determined to be as high as 3.1 × 10^−4^ S m^−1^, and the dependence of the photoconductance on distance and temperature was investigated. Excitation of the Q-band region with a Q-band of SnTPyP^4+^ (550–552 nm) and the band at 714 nm, which is associated with J-aggregation, was responsible for about 34 % of the photoconductive activity of the H_4_${\text{TPPS}}_{4}^{2-}$-Sn(IV)TPyP^4+^ porphyrin nanotubes. The sensor properties of the H_4_${\text{TPPS}}_{4}^{2-}$- Sn(IV)TPyP^4+^ nanotubes in the presence of iodine vapor and salicylate anions down to millimolar range were examined in a chemiresistor sensing mode. We have shown that the porphyrin nanotubes advantageously combine the characteristics of a sensor and a transducer, thus demonstrating their great potential as efficient functional layers for sensing devices and biomimetic nanoarchitectures.

## Introduction

The self-organization of tetrapyrroles in the form of molecular aggregates is known in biological systems for its role in light harvesting, energy transformation, and electron transport. The properties and functions of natural chlorophyll pigments, which in the composition of the chloroplasts carry out the photosynthesis, and hemes, which in the composition of hemoglobin carry out oxygen transport, in the composition of myoglobin—its storage, and in the composition of cytochromes—catalysis of biological redox reactions, inspired wide use of porphyrins and their molecular assemblies as biomimetic materials in systems replicating photosynthesis, electron transport, and enzymatic catalysis. Therefore, the self-assembly of porphyrin macrocycles, whose nanostructures have interesting electronic and optical properties, is being used in search for new nanoscale materials in the field of reversible binding and (photo)catalysis, biomimetic sensors, solar energy conversion, and electrically active components in various nanodevices (Fukuzumi and Imahori, 2008; El-Khouly et al., 2014; Fuhrhop, 2014; Guo et al., 2014; Ou et al., 2014; Zhang et al., 2015; Chen et al., 2016; Koposova et al., 2016a; Mirkovic et al., 2017; Paolesse et al., 2017).

Particular attention has been given in the past to the water-soluble porphyrins. The synthesis of the first J-aggregates of a water-soluble porphyrin, 5,10,15,20-tetra(4-sulphonatophenyl) porphyrin, formed in acidified aqueous solutions, provided the stimulus for the investigations of the self-assembled porphyrin nanostructures (Pasternack et al., 1972; Ohno et al., 1993; Mchale, 2012). Fine-tuning the properties of a metallo-porphyrin complex by varying its different components, such as the molecular skeleton, peripheral substituents, coordinated metal as well as pH and ionic strength of the solutions, allows the creation of self-assembled nanostructures of various shapes with different properties. Self-assembly of porphyrin tectons into nanostructures is controlled by a multiplicity of non-covalent interactions such as hydrogen bonds, electrostatic and π-π interactions, axial coordination, and van der Waals forces (Guldi and Imahori, 2004; Martin et al., 2010, 2013;Würthner et al., 2011).

The electrical and photoconductivity properties of aromatic π-conjugated porphyrin macrocycles and their nanoarchitectures are intensively studied. The π-conjugated porphyrin macrocycles absorb the visible light energy, which may lead to the intermolecular transfer or delocalization of the excitation energy in porphyrin aggregates and arrays making them photoconductive under application of electric field (Weigl, 1957; Golubchikov and Berezin, 1986; Kobayashi et al., 1993; Chou et al., 2000; Drain, 2002; Schwab et al., 2004; Yeats et al., 2008; Kocherzhenko et al., 2009; Friesen et al., 2010; Martin et al., 2010; Riley et al., 2010; Cai et al., 2014; Adinehnia et al., 2016; Koposova et al., 2016b, 2018; Borders et al., 2017). So far, electrical properties of porphyrin nanostructures and the mechanisms of charge transport in their aggregates have not been investigated and explained in details. Photoconductivity was reported in J-aggregates of free-base porphyrins (Schwab et al., 2004; Yeats et al., 2008; Friesen et al., 2010; Riley et al., 2010), whereby most studies so far have dealt with the self-assembled nanorods of 5,10,15,20-tetra(4-sulphonatophenyl) porphyrin. Later on, Adinehnia et al. (2016) and Borders et al. (2017) studied photoconductive properties of porphyrin nanostructures composed of oppositely charged free-base porphyrins: 5,10,15,20-tetra(4-sulfonatophenyl)porphyrin with 5,10,15,20-tetra(N-methyl-4-pyridyl)porphyrin, TPPS_4_:TMPyP, (Adinehnia et al., 2016) or 5,10,15,20-tetra(4-pyridyl)porphyrin, TPPS_4_:TPyP, (Borders et al., 2017).

Martin et al. (2010) described photoconductive microscale clover-shaped structures self-assembled from the water-soluble metalloporphyrins Zn(II)TPPS^4−^ as a donor and Sn(IV)T(N-EtOH-4-Py)P^4+^ as an acceptor. The authors discussed the observed photoconductivity of the nanostructures in terms of exciton delocalization and charge-transfer exciton theory based on a model of the electron-donor-acceptor charge-transfer complex tetrathiafulvalene-tetracyanoquinodimethane (TTF-TCNQ) (Ferraris et al., 1973). By analogy with TTF-TCNQ, the authors proposed an arrangement of individual electron donor, Zn(II)TPPS^4−^, and acceptor, Sn(IV)T(N-EtOH-4-Py)P^4+^, porphyrin molecules in segregated stacks, whereby the charge carriers produced by photoexcitation can move along the stacks, when an electric potential is applied in the direction of the stacks. Formation of heteroaggregates with charge-transfer interactions was also found earlier in the assembly of electron-attracting Au(III) porphyrin and electron-releasing Zn(II) porphyrin (Segawa et al., 1989). Segawa et al. (1992b) reported photoinduced electron-transfer reactions in porphyrin heteroaggregates of the water-soluble Au(III) and Zn(II) porphyrins and formation of the contact radical ion pair as a result of photoexcitation. Recently, photoinduced charge separation has been observed for the ion-pairs obtained from water-soluble cationic and anionic porphyrins ZnTMePyP^4+^ or H_2_TMePyP^4+^ and ZnTPPS^4−^ or H_2_TPPS^4−^ (Natali and Scandola, 2016). In this system, the porphyrins of cationic character were reduced, while the porphyrins of anionic character were oxidized. Collman et al. (2000) reported a series of bis(metalloporphyrin) sandwich complexes as charge-transfer materials and their conductivities. Porphyrins as molecular acceptors form donor-acceptor systems with other molecular donors (Segawa et al., 1992a; Jana et al., 2017). The above studies give an evidence for the electron-donor-acceptor charge-transfer complex mechanism of the photoconductivity of the porphyrin dimers, aggregates, arrays, and nanostructures. However, a better knowledge of the supramolecular non-covalently bonded porphyrin nanoaggregates and their properties is required to understand natural macrocycles assemblies, evaluate their utility as electrical components in nanodevices, and advance the exploitation of their properties, such as excited state delocalization, energy and electron transfer, and photoconductivity in bio-mimicking materials in the fields of photodetectors, solar energy applications, catalysis, and sensors. Earlier, we reported on the self-assembly and photoconductivity of the porphyrin nanostructures in systems of *meso*-substituted Co(III) and Sn(IV) porphyrins as well as *meso*-substituted Co(III) and free-base porphyrins. In this report, we present the morphological, spectral, electrochemical properties, and photoconductivity phenomenon of self-assembled porphyrin nanostructures of m*eso*-tetra(4-sulfonatophenyl)porphyrin and Sn(IV) *meso*-tetra(4-pyridyl)porphyrin, which shows the highest photoconductivity of the last systems.

## Materials and Methods

### Chemicals

**Figure 1:**
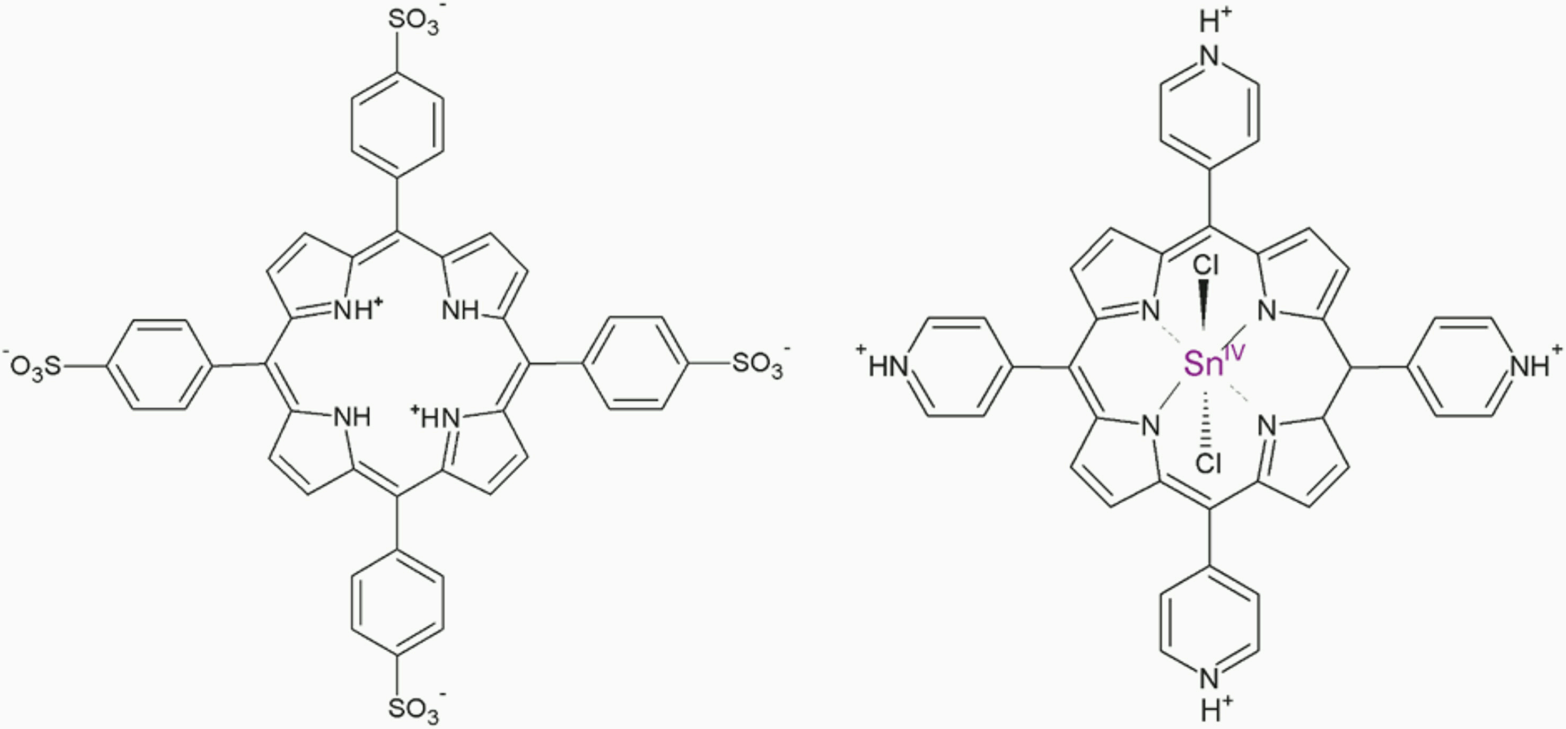
Structures of the porphyrin diacid H_4_${\text{TPPS}}_{4}^{2-}$ and Sn(IV)TPyP^4+^ (pyridyl groups are shown in the protonated state).

Sn(IV) *meso*-tetra(4-pyridyl)porphyrin dichloride and *meso*-tetra(4-sulfonatophenyl)porphyrin dihydrochloride were obtained from Frontier Scientific (>95% purity) and used as received, (Figure 1). All other chemicals were from Sigma-Aldrich. Diamond (1 μm) and alumina (0.05 μm) polishing suspensions and the corresponding polishing pads were from ALS Co. All solutions were prepared using distilled water. Sodium hydroxide and hydrochloric acid solutions were used to adjust the pH.

### Nanotube Synthesis

The porphyrin nanotubes were prepared using equal volumes of the 10 μM stock solutions of individual porphyrins (an equimolar, 1:1 concentration ratio) and adjusting the pH of the mixture to pH 2, 6, and 10.5. The formation of colloids started immediately at pH 2, while at pH 6 and 10.5 no nanostructure formation was observed. The nanostructures were also prepared using 1:5 and 5:1 concentration ratios of Sn(IV)TPyP^4+^ : H_4_${\text{TPPS}}_{4}^{2-}$ in solutions at pH 2. The colloidal solutions remained in the dark for 5 days. To prepare the stock solutions, Sn(IV)TPyP^4+^ was dissolved in 0.02 M HCl, while H_2_${\text{TPPS}}_{4}^{4-}$/H_4_${\text{TPPS}}_{4}^{2-}$ (TPPS_4_) was dissolved in water.

### Characterization of the Structure and Composition

The nanostructures were characterized by scanning electron microscopy using a Gemini 1550 VP SEM (Carl Zeiss, Jena, Germany). To prepare the samples for SEM, 50 μL of the nanostructures solutions were dropped on the Si substrates, and the samples were left to dry for 12 h. After that, the samples were washed with 0.01 M HCl, water, and left to dry. The transmission electron microscopy (TEM) images were obtained using a FEI Tecnai G2 F20 microscope operated at 200 kV. EDX analysis was performed using a JEOL 840 A. The samples of the nanostructures for the EDX analysis were concentrated in a 0.02 M HCl by centrifugation (10,000 rpm 7 min) and washing several times until a transparent supernatant solution was obtained to remove not self-assembled porphyrins. Fifty microliter of the nanostructures solutions were dropped on the Si/SiO_2_ substrates, and the samples were left to dry. This procedure was repeated four times. After that, the samples were washed with 0.01 M HCl, water, and left to dry. The AFM measurements of the nanostructures on the Si/SiO_2_ substrates were performed using a MultiMode scanning probe system (Bruker) in a tapping mode. The samples were prepared as described above for the SEM samples.

The optical spectra were obtained on a PerkinElmer Lambda 900 spectrometer. Quartz cuvettes with a path length of 10 and 5 mm were used. Individual porphyrin solutions for spectra measurements were prepared by dissolving Sn(IV)TPyP^4+^ in acidified water (pH = 2.0) and H_4_${\text{TPPS}}_{4}^{2-}$ in water. The samples of the nanostructures for the UV-vis spectrometry were concentrated in a 0.02 M HCl by centrifugation (10,000 rpm 7 min) and washing several times until a transparent supernatant solution was obtained to remove not self-assembled porphyrins.

The ratio of S:N atoms was found using an elemental analysis, which determines the absolute element content. The samples of the nanorods for the elemental analysis were prepared by centrifugation (10,000 rpm 7 min) and washing of the nanorods solutions with subsequent drying of the collected sediments and analyzed by the “vario El cube” (Elementar) with a thermal conductivity detector.

### Electrochemical Methods

Electrochemical experiments were performed with a potentiostat (Autolab PGSTAT100, The Netherlands) controlled by the Nova 2.1 software. The experimental setup for recording the cyclic voltammograms included a three-electrode electrochemical cell. A coiled platinum wire was used as a counter electrode. A glassy carbon electrode (BASi Inc.) with a diameter of 3 mm was used as a working electrode. The potentials were controlled relative to a double junction Ag/AgCl, Metrohm, Ag | AgCl | KCl 3 M::0.5 M KCl reference electrode. The solution in the bridge of the reference electrode was replaced after each measurement to avoid contaminations of the electrochemical cell. Measurements were carried out under Ar in the dark at room temperature (21 ± 1°C). Glassy carbon electrodes were cleaned before each electrochemical measurement as described below: the electrodes were polished with diamond (1 μm) and then alumina (0.05 μm) slurry on the respective polishing cloths. Immediately after polishing, the electrodes were rinsed with distilled water and sonicated in a mixture of water and ethanol for about 10 s to remove polishing residues from the electrode surface. The electrodes were then thoroughly rinsed with distilled water and dried with nitrogen.

### Electrophotoresponse Measurements

To measure the (photo)conductance of the porphyrin nanorods, two types of thin-film gold electrodes were used: gold electrode pairs with an electrode gap of 400 nm, (Figure S1), and interdigitated electrode arrays with a 2 μm spacing between the electrode lines and an electrode line thickness of 2 μm. The electrodes were produced on a Si substrate with a silicon dioxide layer of 1,000 nm thickness using electron beam lithography, lift-off process, and thin-film technologies in an ISO 5 cleanroom as described in detail in Muratova et al. (2016). Thin metal layers of titanium for adhesion (10 nm) and gold (50 nm) were deposited by means of an electron beam evaporation using a Pfeiffer PLS 500 equipment. After fabrication, the electrodes were cleaned in acetone and isopropanol. After that, the electrodes were treated with oxygen plasma (≪Plasma system FEMTO≫). Finally, the electrodes were rinsed with ethanol and distilled water. Two microliter of the porphyrin nanostructures solution was pipetted onto the electrodes and the samples were allowed to dry in the dark for 2 h. The samples were then rinsed with 0.01 M HCl, water, and allowed to dry for 24 h. The measurements were also performed using an equimolar solution of TPPS_4_ and Sn(IV)TPyP^4+^ of pH 6 instead of the nanostructure solution. The device was prepared in a similar way as for the nanostructures, namely, 2 μL of the solution of porphyrins was pipetted onto the electrodes and the device was allowed to dry in the dark for 2 h. The device was then rinsed with water and allowed to dry for 24 h.

The (photo)conductance was recorded with a Keithley 4200 SCS semiconductor analyzer using a two-probe configuration. Applied potential was 0.5 V. The gold electrodes were connected to the external circuit by contacting the bond pads with tungsten needles. A 150 W xenon arc lamp assembled with an AM1.5 filter (Oriel Instruments, Model No. 6255) and a visible light filter (390–630 nm) was used for photoexitation. The light intensity of the beam focused on the sample during experiments was measured using a photodetector (CAS140CT-154 Kompakt-Array-Spektrometer model UV-vis-NIR, Instrument Systems) and equaled 29 mW cm^−2^.

## Results and Discussion

### Nanotube Morphology

**Figure 2:**
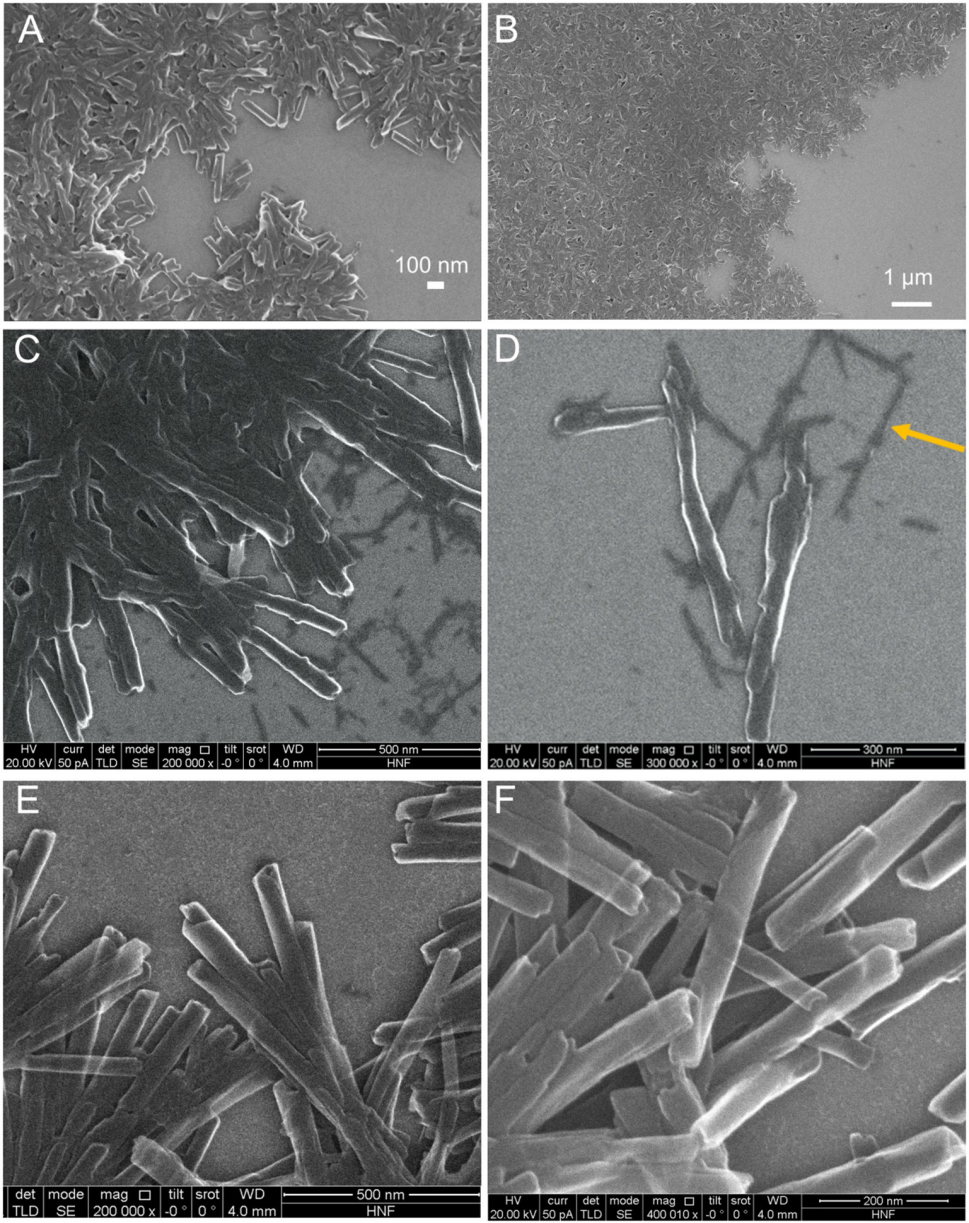
SEM images of the H_4_${\text{TPPS}}_{4}^{2-}$-Sn(IV)TPyP^4+^ porphyrin nanostructures formed by the self-assembly in solutions at pH = 2.0 and Sn(IV)TPyP^4+^ and H_4_${\text{TPPS}}_{4}^{2-}$ taken in a 1:1 concentration ratio **(A,B)**, Sn(IV)TPyP^4+^ and H_4_${\text{TPPS}}_{4}^{2-}$ taken in a 1:5 concentration ratio **(C,D)**, the arrow shows the second type of nanostructures formed in this system, which are thinner nanorods, also visible in image **(C)**, and Sn(IV)TPyP^4+^ and H_4_${\text{TPPS}}_{4}^{2-}$ taken in a 5:1 concentration ratio **(E,F)**.

**Figure 3:**
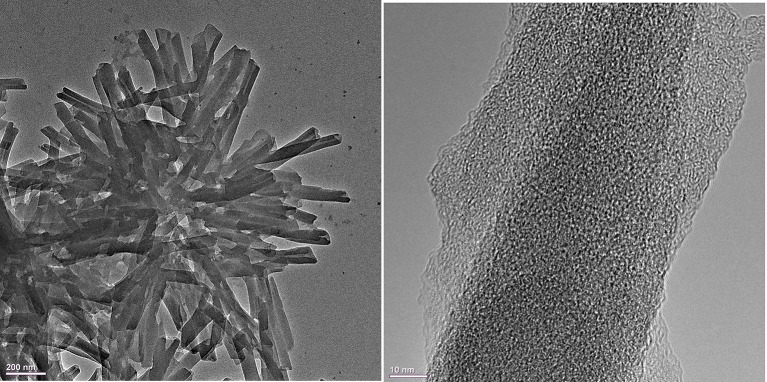
TEM images of the H_4_${\text{TPPS}}_{4}^{2-}$ –Sn(IV)TPyP^4+^ porphyrin nanostructures formed in equimolar porphyrin solutions at pH = 2.0 by the self-assembly reaction.

**Figure 4:**
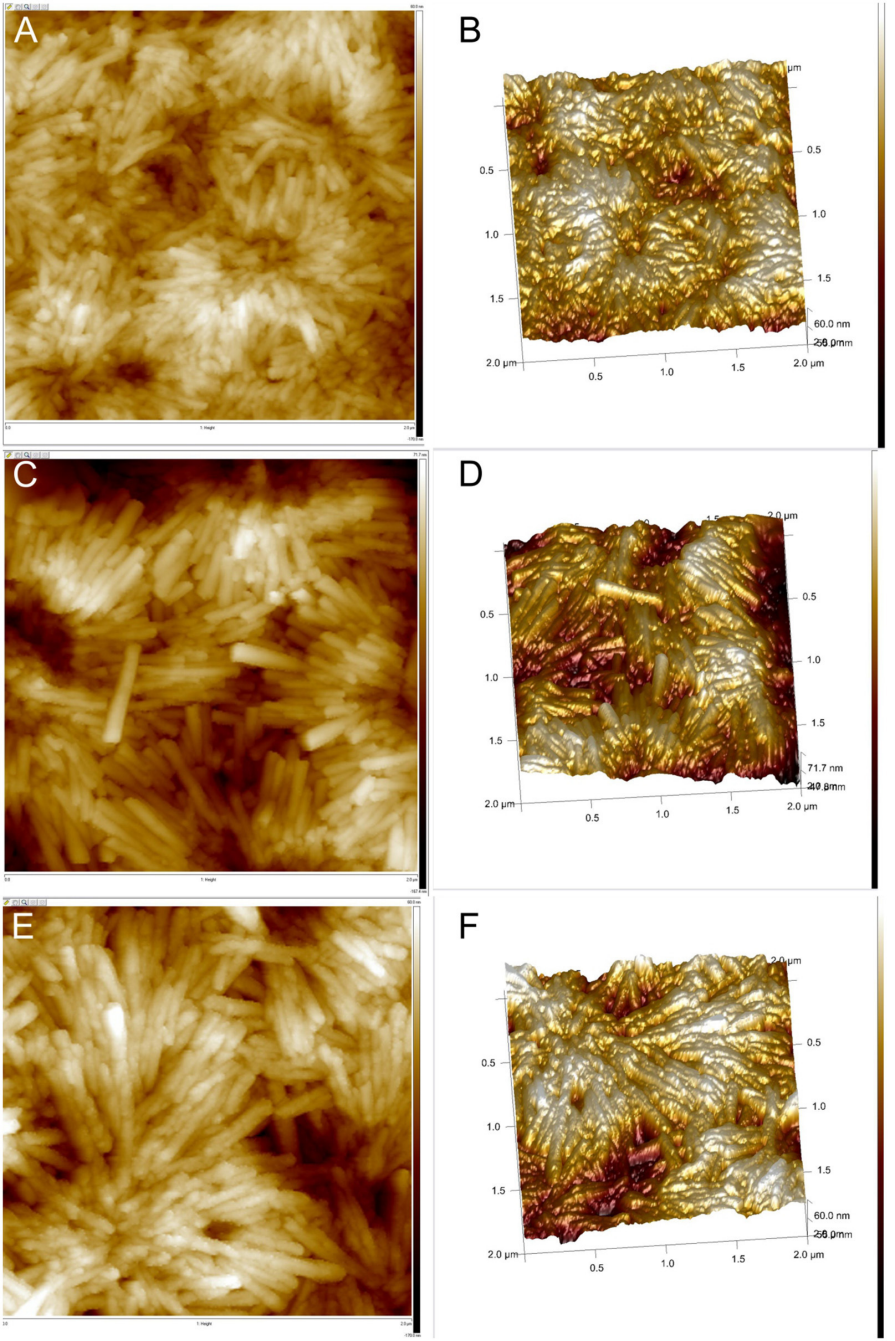
AFM images of the H_4_${\text{TPPS}}_{4}^{2-}$-Sn(IV)TPyP^4+^ porphyrin nanostructures formed by the self-assembly in solutions at pH = 2.0 and Sn(IV)TPyP^4+^ and H_4_${\text{TPPS}}_{4}^{2-}$ taken in a 1:1 concentration ratio **(A,B)**, Sn(IV)TPyP^4+^ and H_4_${\text{TPPS}}_{4}^{2-}$ taken in a 1:5 concentration ratio **(C,D)**, and Sn(IV)TPyP^4+^ and H_4_${\text{TPPS}}_{4}^{2-}$ taken in a 5:1 concentration ratio **(E,F)**.

Sn(IV)TPyP^4+^ and H_4_${\text{TPPS}}_{4}^{2-}$ solutions produced brown precipitates in a self-assembly reaction at pH 2. It should be noted that that for a concentration ratio of 5:1 (Sn(IV)TPyP^4+^ : H_4_${\text{TPPS}}_{4}^{2-}$), a very small amount of the nanostructures is formed in a self-assembly reaction, which makes them not very suitable for practical use. SEM, TEM, and AFM analyses revealed that these two porphyrins form the tubular nanostructures, (Figures 2–4) and (Figures S2, S3).

TEM images show that the tubular nanostructures are hollow with open ends. These structural details are clearer at the edges of the round formed conglomerates, because the material layers overlap at the places with a higher density of nanotubes. Additionally, the open ends of the nanotubes can be seen in some of the SEM images, e.g., (Figure S2B). The nanotubes have a length of 0.4–0.8 μm, an inner diameter of 7–15 nm, and an outer diameter of 30–70 nm, (Figures 2–4) and (Figures S2, S3).

Interestingly, SEM and AFM investigations indicated a presence of two types of the nanostructures in a system, where the porphyrins were taken in a 1:5 (Sn(IV)TPyP^4+^: H_4_${\text{TPPS}}_{4}^{2-}$) concentration ratio, (Figures 2C,D, 4C,D) and (Figures S2, S4). One kind of the nanostructures is similar to the nanostructures in the systems, where the porphyrins were taken in 1:1 and 5:1 (Sn(IV)TPyP^4+^ and H_4_${\text{TPPS}}_{4}^{2-}$) concentration ratios, while the presence of much thinner nanorods of about 10 nm diameter and patches (unrolled pieces of nanorods) of about 6 nm thickness is observed in a 1:5 (Sn(IV)TPyP^4+^ : H_4_${\text{TPPS}}_{4}^{2-}$) system, (Figures 2C,D) and (Figures S2, S4). We assume that these thinner and segregated nanostructures are H_4_${\text{TPPS}}_{4}^{2-}$ self-assembled nanorods formed due to the excess of this porphyrin because of the structural and optical absorption similarity (see below). The thin nanorods and patches were observed to be located under the thicker Sn(IV)TPyP^4+^-H_4_${\text{TPPS}}_{4}^{2-}$ nanotubes and on the edges of the layers formed by the thick Sn(IV)TPyP^4+^-H_4_${\text{TPPS}}_{4}^{2-}$ nanotubes, (Figures 2C,D) and (Figure S2). One may conclude that the excess of H_4_${\text{TPPS}}_{4}^{2-}$ results in a larger dispersion of the nanostructures, which is also supported by the AFM investigation below, producing at least two types of the nanostructures. These segregated thin nanorods and patches do not form, however, continuous layers like the thick Sn(IV)TPyP^4+^-H_4_${\text{TPPS}}_{4}^{2-}$ nanotubes form.

An interesting feature of the nanostructures self-assembled in a system, where the porphyrins were taken in a 5:1 (Sn(IV)TPyP^4+^ : H_4_${\text{TPPS}}_{4}^{2-}$) concentration ratio, in comparison with other two systems at pH 2 was a transparency of the nanotubes, (Figures 2E,F) which is probably related to their thickness or non-dense structure.

AFM analysis showed that the nanostructures self-assembled from equimolar solutions of two porphyrins produced smoother layers, (Figures 4A,B), while the nanostructures obtained from 1:5 (Sn(IV)TPyP^4+^ : H_4_${\text{TPPS}}_{4}^{2-}$) solutions formed more rough layers, (Figures 4C,D), which might be explained by the presence of the nanostructures of two types. Additionally, AFM showed a different morphology of the nanotube surfaces formed in a system, where the porphyrins were taken in a 5:1 (Sn(IV)TPyP^4+^ : H_4_${\text{TPPS}}_{4}^{2-}$) concentration ratios. One can observe a nodular or twisted surfaces of the nanotubes, (Figures 4E,F). This feature requires, however, further investigations.

The nanotubes in the TEM images appear in round formed agglomerates, being connected together by one edge and diverging in different directions from one place. The appearance of the round nanostructure conglomerates may emerge either in the solution during synthesis after the formation of individual nanotubes which then stick together or after deposition of the nanotubes on a solid substrate and subsequent water evaporation. Thus, SEM, TEM, and AFM analyses show that the structural features of the H_4_${\text{TPPS}}_{4}^{2-}$ –Sn(IV)TPyP^4+^ binary nanotubes differ essentially from those of the self-assembled H_4_${\text{TPPS}}_{4}^{2-}$ nanorods. The latter are well-segregated nanorods with an essentially smaller outer diameter of about 10–15 nm, as it is shown in Figure S5. The smaller nanostructures in the system, where the porphyrins in the solution were taken in a concentration ratio of 1:5 (Sn(IV)TPyP^4+^ : H_4_${\text{TPPS}}_{4}^{2-}$), were probably the result of self-assembly mainly H_4_${\text{TPPS}}_{4}^{2-}$ because of its excess. A synergy of various intermolecular interactions and structural features of individual porphyrin tectons (Agranovich and Bassani, 2003; Guldi and Imahori, 2004; Medforth et al., 2009; Koposova et al., 2016b, 2018) are responsible for a great multiplicity of the observed 1D to 3D geometries of porphyrin molecular aggregates formed by self-assembly.

The self-assembly of the H_4_${\text{TPPS}}_{4}^{2-}$ : SnTPyP^4+^ porphyrin nanotube (2.4:1 mol mol^−1^) was first shown by Shelnutt and co-workers (Wang et al., 2004). The self-assembly was highly pH dependent, since the protonation states of the porphyrin molecules determined the balance of molecule charges for the assembly (Wang et al., 2004; Franco et al., 2010). At pH = 2.0, partially dissociated sulfonato-groups (pK_a_ = 2.6 for benzenesulfonic acid), a protonated center of H_4_${\text{TPPS}}_{4}^{2-}$ (pK_a_ = 4.9), and protonated pyridyl groups of Sn(IV)TPyP^4+^ (pK_a_ = 5.2 for pyridine) participate in the molecular assembly. Influence of the pH on the porphyrin molecular assembly was also documented in the study of the Sn(IV)TPPS_4_-Co(III)TPyP nanostructures, where the Sn:Co atomic ratio was found to be 1:1.15 at pH 2.7 and 1:3 at pH 4.8 (Koposova et al., 2016b). These molecule ratios were due to the neutralization of anionic and cationic porphyrin species at different pH. Accordingly, pH plays an important role in the self-assembly, since it determines the electrostatic interactions of the porphyrin tectons.

**Figure 5:**
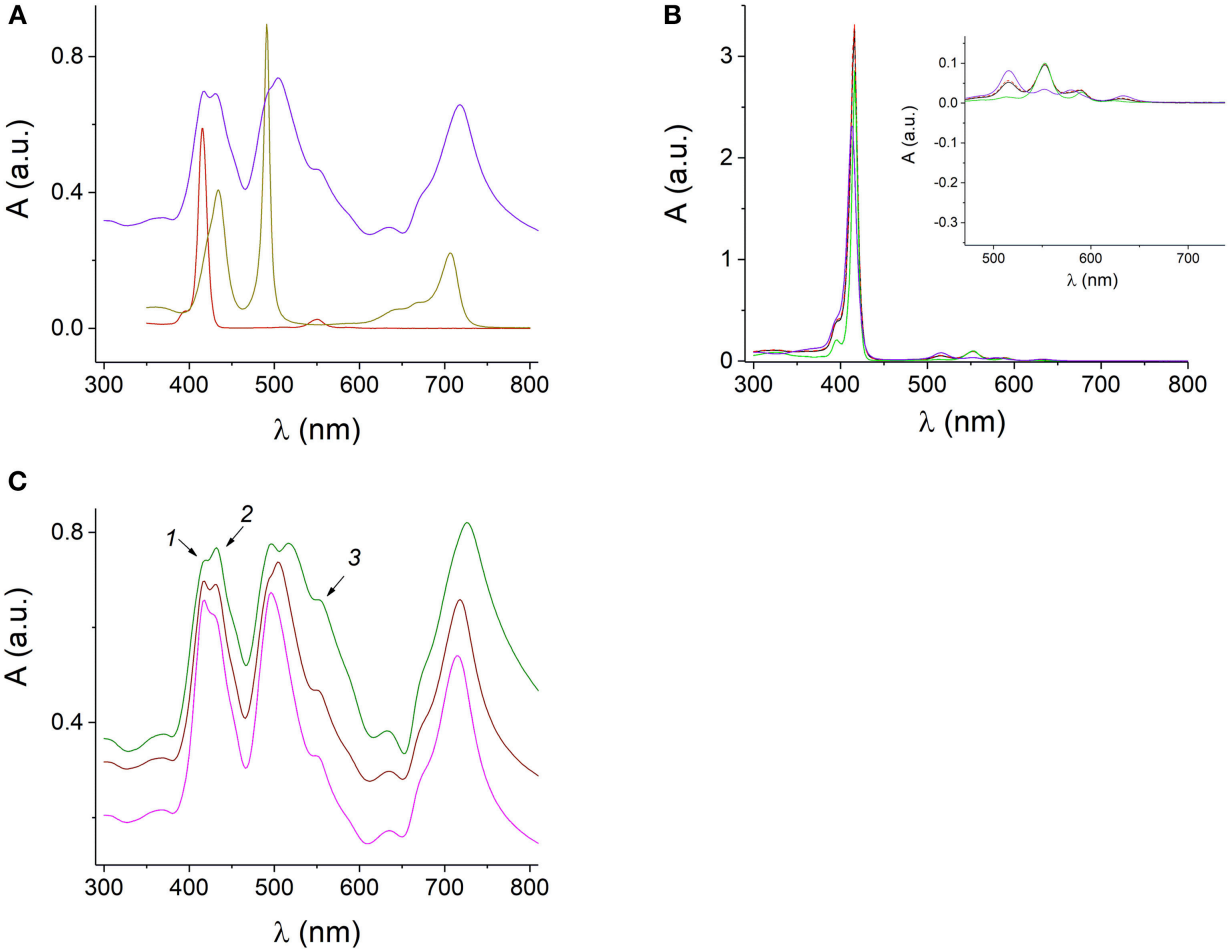
UV-visible absorption spectra of the protonated H_4_${\text{TPPS}}_{4}^{2-}$ and H_4_${\text{TPPS}}_{4}^{2-}$ J-aggregates at pH 0.94 (dark yellow), 1 μM SnTPyP^4+^ (dark red), and H_4_${\text{TPPS}}_{4}^{2-}$-SnTPyP^4+^ nanotubes (blue) at pH 2 **(A)**. Spectra of the mixed equimolar solutions of TPPS_4_ and SnTPyP^4+^ porphyrins at pH 6 (red dashed line) and pH 10.5 (black line) and the spectra of the corresponding porphyrins at pH 10.5: SnTPyP at pH 10.5 (green) and deprotonated TPPS_4_ at pH 10.5 (violet). The formation of nanostructures is not observed. The insert in **(B)** shows an enlarged long-wavelength region **(B)**. Spectra of the SnTPyP^4+^-H_4_${\text{TPPS}}_{4}^{2-}$ nanotubes self-assembled in solutions at pH 2 and SnTPyP^4+^ and H_4_${\text{TPPS}}_{4}^{2-}$ taken in concentration ratios of 1:5 (green line), 1:1 (brown), and 5:1 (pink). The arrows indicate the spectral features characteristic for the absorption of individual porphyrins **(C)**.

In our study, TPPS_4_ and Sn(IV)TPyP^4+^ taken in a 1:1 concentration ratio did not self-assembly into the nanostructures at pH 6 and 10.5, where TPPS_4_ is predominantly in its deprotonated H_2_${\text{TPPS}}_{4}^{4-}$ form. This indicates that the presence of the protonated center of H_4_${\text{TPPS}}_{4}^{2-}$ and its assembly into the slipped face-to-face configuration is a driving force for the self-assembly process, while the Sn(IV)TPyP^4+^ tectons, which do not produce the nanostructures by its own, co-assembly in this self-assembly process. In these cases, the optical absorption spectra, (Figure 5), of Sn(IV)TPyP^4+^ and TPPS_4_ taken in 1:1 concentration ratio at pH 6 and 10.5 are practically overlap of the optical absorption spectra of the individual porphyrins at the same pH and are not indicative for the formation of new species. In addition, as it was mentioned earlier, a very small amount of nanostructures was formed if the initial molar ratio of H_4_${\text{TPPS}}_{4}^{2-}$ : SnTPyP^4+^ in solution was 1:5, pH 2. Altogether, these observations indicate that H_4_${\text{TPPS}}_{4}^{2-}$ represents the driving force for self-assembly in this porphyrin couple, while SnTPyP^4+^ is rather included due to its cationic nature.

The UV-visible absorption spectrum of the H_4_${\text{TPPS}}_{4}^{2-}$ nanorods demonstrates a sharp J-band at 491 nm red-shifted from the monomer absorption and distinguished by its narrowness and high extinction coefficient together with a band at 706 nm in a Q-region typical for the J-aggregates of H_4_${\text{TPPS}}_{4}^{2-}$, (Figure 5).

Absorption spectrum of the H_4_${\text{TPPS}}_{4}^{2-}$-SnTPyP^4+^ nanotubes prepared using different molar ratios of the porphyrins in the solutions at pH 2 in our study, Figures 5A,C, indicates presence of the J-aggregates in the Sn(IV)TPyP^4+^-H_4_${\text{TPPS}}_{4}^{2-}$ self-assembly according to the characteristic absorption bands at λ = 500–516 nm and λ = 714–726 nm. The bands are red-shifted and broadened compared to the absorption bands of the H_4_${\text{TPPS}}_{4}^{2-}$ nanorods (dark yellow line), (Figures 5A,C). Interestingly, that in the case of a system, where SnTPyP^4+^ and H_4_${\text{TPPS}}_{4}^{2-}$ porphyrins were taken in a concentration ratio of 1:5 (green line), (Figure 5C), the J-band at about 500–516 nm is splitted, which may support existence of two types of nanostructures in this system, as it was discussed above. The presence of J-aggregate bands indicates that excitons may be delocalized over multiple molecules (Torres and Bottari, 2013). Thin nanotubes formed by self-assembly of the metal-free porphyrin H_4_${\text{TPPS}}_{4}^{2-}$ into slipped face-to-face columnar arrangements as well as the mechanism of their formation have been presented and discussed in a series of studies (Ohno et al., 1993; Maiti et al., 1995; Würthner et al., 2011; Mchale, 2012). Inclusion of the hexacoordinated Sn(IV)TPyP^4+^ metalloporphyrin with a heavy metal results in thicker nanotubes with larger sizes saving stack configuration in the assembly and interrupting the usual dipole coupling that lead to broadening of J-bands (Franco et al., 2010). Unlike H_4_${\text{TPPS}}_{4}^{2-}$, Sn(IV)TMPyP^4+^ does not form homoaggregates on its own (George et al., 2010). This may be explained by the presence of the obligate axial ligands of the Sn(IV) porphyrin, which is expected to inhibit macrocycle stacking due to the lack of a cationic center (Ohno et al., 1993; Franco et al., 2010). The Sn^4+^ ion with the axially coordinated Cl^−^ ions hinder the face-to-face geometry, preventing its own J-aggregates but allowing electrostatic interaction between H_4_${\text{TPPS}}_{4}^{2-}$ and SnTPyP^4+^ via the cationic and anionic peripheral groups (Rosaria et al., 2008), which is another force of the assembly process. In Koposova et al. (2016b), a cationic center of the H_4_${\text{TPPS}}_{4}^{2-}$ dianion was replaced with a metal cation, which resulted in a network-like nanostructures prepared by self-assembly of two metalloporphyrins, Sn(IV)TPPS_4_-Co(III)TPyP, instead of well-formed nanorods characteristic for the H_4_${\text{TPPS}}_{4}^{2-}$ self-assembly. Thus, the central metal affects the assembly dimension (Rosaria et al., 2008), which is useful for modulating the aggregate size and properties. Based on previous literature and our experiments above, we assume that the main driving force of self-assembly in this porphyrins pair is assembly of the TPPS_4_ dianion, H_4_${\text{TPPS}}_{4}^{2-}$, with a slipped face-to-face stacking with inclusion of a six-coordinated tin porphyrin, which do not form aggregates by its own. However, exact relative molecular arrangement of both porphyrins in these nanostructures remains under discussion (Wang et al., 2004; Franco et al., 2010).

**Figure 6:**
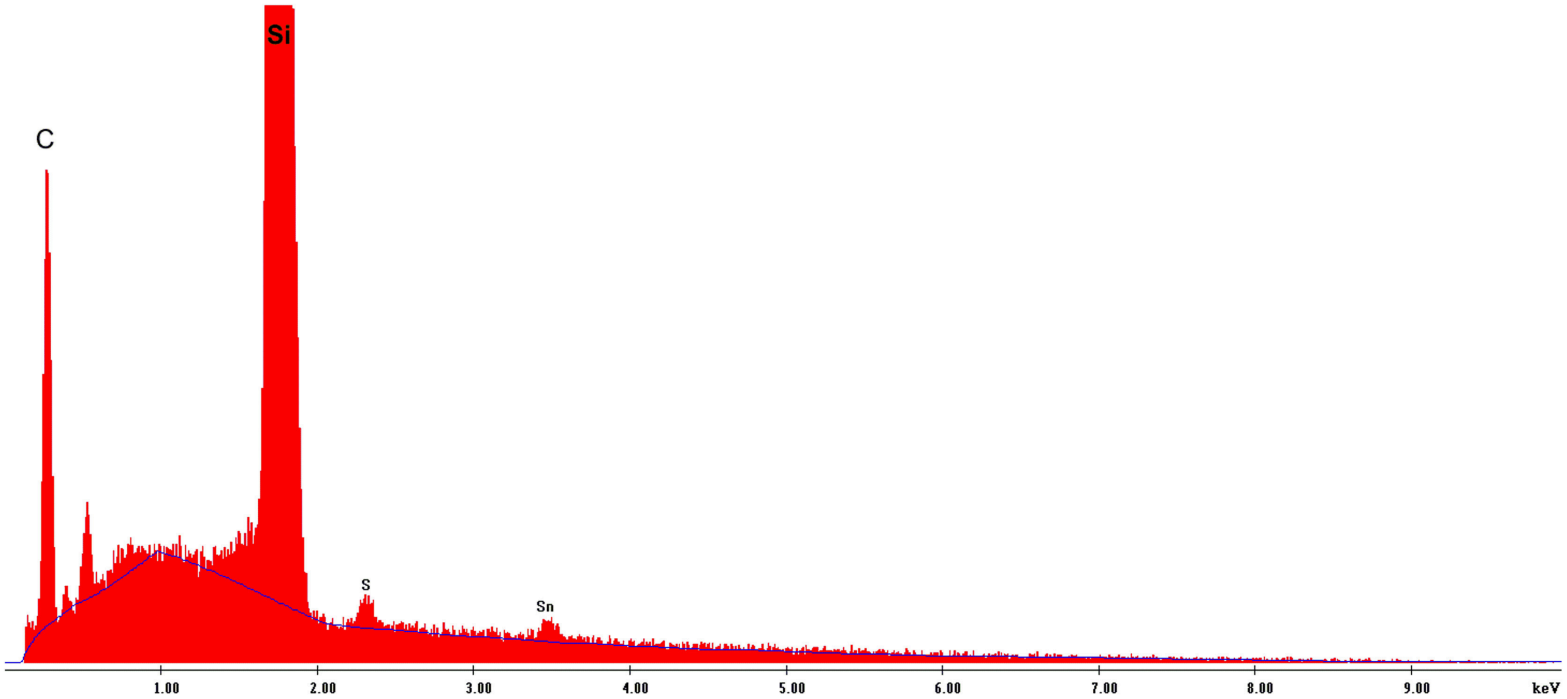
EDS analysis of the H_4_${\text{TPPS}}_{4}^{2-}$-Sn(IV)TPPyP^4+^ nanotubes formed in an equimolar solution at pH = 2.0.

In the present study, EDX spectroscopy showed a presence of both porphyrins (according to the presence of both sulfur and tin elements) in the self-assembled nanotubes, (Figure 6 and Figure S6). Because of the poor accuracy of quantitative EDX analysis, we used chemical elemental analysis to estimate the presence of both porphyrins in the nanotubes. Since a very small amount of the nanostructures is formed in a 5:1 (Sn(IV)TPyP^4+^ : H_4_${\text{TPPS}}_{4}^{2-}$) solution, and system 1:5 (Sn(IV)TPyP^4+^ : H_4_${\text{TPPS}}_{4}^{2-}$) produced at least two different kinds of nanostructures, we performed an elemental analysis of the nanostructures self-assembled from the equimolar porphyrin solutions at pH 2, Figure S7, which indicated a H_4_${\text{TPPS}}_{4}^{2-}$ : SnTPyP^4+^ molar ratio in the nanostructures of 2:1.

### Photoconductivity

**Figure 7:**
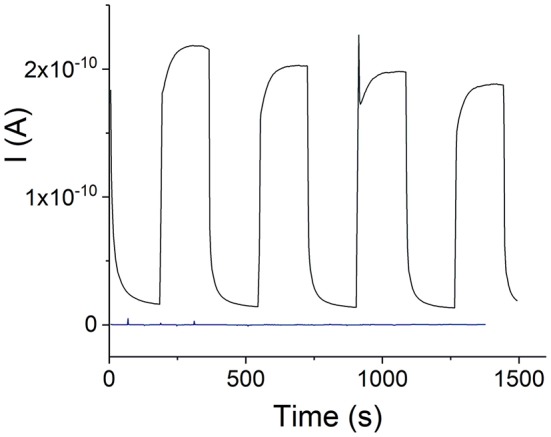
Visible light photoresponse of the H_4_${\text{TPPS}}_{4}^{2-}$-SnTPyP^4+^ nanotubes prepared in an equimolar solution at pH 2, electrode gap 400 nm, V_app_ = 0.5 V. A dark blue curve shows a visible light photoresponse of a device with no nanostructures. The device was prepared by dropcasting an equimolar solution of TPPS_4_ and SnTPyP^4+^ (pH 6) onto the chip as described in experimental section Electrophotoresponse Measurements.

The electrophotoresponse of the H_4_${\text{TPPS}}_{4}^{2-}$-SnTPyP^4+^ porphyrin nanotubes was measured in the dark (dark current) and after photoexcitation with visible light, (Figure 7). Photoexcitation resulted in a photocurrent in case of the H_4_${\text{TPPS}}_{4}^{2-}$-SnTPyP^4+^ porphyrin nanotubes as one can see in Figure 7. Photoresponse of a device, where an equimolar solution of TPPS_4_ and SnTPyP^4+^ porphyrins (pH 6) was dropcasted onto the device and prepared for the measurements in the same way as for the nanostructures (see section Electrophotoresponse Measurements) is also shown. However, no photocurrent was observed in this case probably due to the absence of a permanent layer of porphyrins in the gap between the contact electrodes in the conditions of experiments and a lower photoconductivity of the individual porphyrin molecular layers.

After subtracting the dark current, the apparent photoconductivity of the H_4_${\text{TPPS}}_{4}^{2-}$ –Sn(IV)TPyP^4+^ nanotubes at 23 °C was estimated as (3.1 ± 0.9) × 10^−4^ S m^−1^ (*n* = 5). Slightly lower and less reproducible value, (2.2 ± 1.0) × 10^−4^ S m^−1^, was found for the nanostructures prepared in a solution with a Sn(IV)TPyP^4+^ : H_4_${\text{TPPS}}_{4}^{2-}$ concentration ratio of 1:5. We assume that this might be due to the presence of several types of the smaller nanostructures formed by H_4_${\text{TPPS}}_{4}^{2-}$, which do not form a continuous layer over hundreds of nm distances, (Figures 2C,D and Figure S5), and contact area with the metal electrodes. The latter value is thus not a characteristic of one type of nanostructures, but rather characterizes a nonhomogeneous mixture of the nanostructures obtained in this system. Amounts of the nanostructures prepared in a solution with a Sn(IV)TPyP^4+^ : H_4_${\text{TPPS}}_{4}^{2-}$ concentration ratio of 5:1 was practically very small for the accurate measurements of photoconductivity, giving a similar approximate value of (1.8 ± 0.8) × 10^−4^ S m^−1^ (*n* = 2). Identical spectral features of the nanostructures prepared at pH 2, (Figure 5C), are in agreement with similar values of photoconductivities in these systems.

The value of the apparent photoconductivity of the H_4_${\text{TPPS}}_{4}^{2-}$-Sn(IV)TPyP^4+^ nanotubes is lower than for the porphyrin-acetylene-thiophene polymer wires synthesized in Li et al. (2004), which, had, however, covalent nature, (Table 1). On the other hand, the apparent photoconductivity of the H_4_${\text{TPPS}}_{4}^{2-}$-Sn(IV)TPyP^4+^ nanotubes is higher by several orders of magnitude than for similar systems, (Table 1), and corresponds to the conductivity region of semiconductors (Kobayashi et al., 1993; Naarman, 2012). As can be seen in Table 1, the photoconductivities of the porphyrin films and porphyrin self-assembled nanostructures were demonstrated in a range of 10^−10^-10^−3^ S m^−1^ (Golubchikov and Berezin, 1986).

**Table 1:** Conductivity and photoconductivity of different self-assembled and covalently bonded porphyrin systems.

**Porphyrin system**	**σ, S m^**−1**^**	**References**
Tetraphenylporphine (TPP) film^a^	1.5 × 10^−9^	Weigl, 1957
Oxygen-doped (air) ZnTPP thin film^b^	< 10^−9^	Kobayashi et al., 1993
Charge-transfer complexes of Lanthanide(III) bis(porphyrin) sandwich complexes of Gd and Lu and Zr(IV) bis(porphyrin) sandwiches^b^	10^−5^-8 × 10^−3^	Collman et al., 2000
Cu-porphyrin Langmuir-Blodgett film^b^	2.54 × 10^−5^ (*x* axis) and 4.71 × 10^−7^ (*y* axis)	Zhang et al., 1995
Porphyrin-acetylene-thiophene polymer wires^b^	6 × 10^−1^	Li et al., 2004
P(V)-porphyrin(electron acceptor)-oligothiophene(electron donor) polymers^b^^,^^c^	1.2 × 10^−7^-5.1 × 10^−6^	Segawa et al., 1992a
Porphyrin polymer with oligophenylenevinylene		Jiang et al., 1997
bridge undoped^b^	< 10^−10^	
after doping^b^	10^−4^	
Nanofilaments of tetra-*meso*-amidophenyl substituted porphyrin^a^^,^^d^ 90–400 W m^−2^, bias 1 V	up to 100 × 10^−12^ m Ω^−1^ W^−1^	Schall et al., 2015
*meso*-tri(4-sulfonatophenyl)monophenylporphine, TPPS_3_, nanotapes ^a^^,^^d^	5 × 10^−8^ m Ω^−1^ W^−1^	Yeats et al., 2008
Chlorophyll-a^a^^,^^e^	(0.2–1.0) × 10^−8^	Jones et al., 1983
Protoporphyrin IX dimethyl ester	1.5 × 10^−5^	
TPyP:TSPP crystalline rods^a^^,^^f^	9.3 × 10^−8^	Borders et al., 2017
TMPyP:TSPP crystalline rods ^a^^,^^g^	4.0 × 10^−10^	Adinehnia et al., 2016
H_2_TMPyP:Cu(II)TSPP^a^^,^^h^	7.7 × 10^−8^	Borders et al., 2018
Cu(II)TMPyP:H_2_TSPP	1.1 × 10^−8^	
H_2_TMPyP:Ni(II)TSPP	7.7 × 10^−9^	
Ni(II)TMPyP: H_2_TSPP	6.3 × 10^−9^	
Sn(IV)TPPS_4_-Co(III)TPyP nanostructures^a^^,^^i^	6 × 10^−7^	Koposova et al., 2016b
Co(III)TPyP-H_4_${\text{TPPS}}_{4}^{2-}$ porphyrin nanostructures^a^^,^^i^	5 × 10^−5^	Koposova et al., 2018
Sn(IV)TPyP-H_4_${\text{TPPS}}_{4}^{2-}$ porphyrin nanotubes^a^^,^^i^	3.1 × 10^−4^	This work

a *Photoconductivity*.

b *Conductivity*.

c *In polymers the conductivity was enhanced by the photoirradiation. In the case of photoirradiation by 500 W Xe lamp through UV and IR cut-off filters, the enhancement was > 3 fold (Segawa et al., 1992a)*.

d *The photoconductivity in this study is defined as the conductivity divided by the light intensity*.

e *Photoconductivity parallel to the plane of the multilayer, in-plane photoconductivity, 50 W m^−2^ of white light, 5 V bias*.

f *At 405 nm excitation, 10 kW m^−2^, 2 V bias*.

g *At 445 nm excitation, 10 kW m^−2^, 2 V bias*.

h *At 445 nm excitation, 1.0 W cm^−2^, 5 V bias*.

i *Visible light, Xe lamp, 0.25 kW m^−2^, bias 0.5 V*.

Figure 6A shows that the photocurrent of the self-assembled H_4_${\text{TPPS}}_{4}^{2-}$-Sn(IV)TPyP^4+^ nanotubes decreases over the μm distances. The dependence of the photroconductance of the H_4_${\text{TPPS}}_{4}^{2-}$ –SnTPyP^4+^ nanostructures on temperature was investigated in the range from 23 to 70°C, (Figure 6B). Increasing the temperature resulted in a decrease in the photocurrent, dσ/dT < 0. At the highest temperature, 70 °C, a decrease in the dark current was also observed. The metal-like character of the dependence of the photoconductivity of the H_4_${\text{TPPS}}_{4}^{2-}$ –SnTPyP^4+^ nanotubes on temperature was also observed earlier for the H_4_${\text{TPPS}}_{4}^{2-}$ –Co(III)T(4-Py)P self-assembled nanostructures (Koposova et al., 2018). Furthermore, the photoconductivity of the Sn(IV)TPPS_4_-CoTPyP nanostructures was characterized by the decrease in both photocurrent and dark current at the elevated temperature (Koposova et al., 2016b). The observed dependence may be explained by overcoming the energy of intermolecular interactions with increased disorder, as well as the recombination of electrons and holes at elevated temperatures. A similar metal-like character of the conductance was also observed in a TTF-TCNQ complex in a narrow range of temperature (Ferraris et al., 1973) and arrayed iodine-doped metallo-macrocycles (Schramm et al., 1980; Hoffman and Ibers, 1983; Golubchikov and Berezin, 1986). After cooling down to the initial temperature, the conductance properties recovered and photoconductance even increased slightly, (Figure 6B) (dotted line). This indicates that the destruction of the nanostructure assembly does not occur during heating.

Photoinduced charge transfer in self-assembled porphyrin nanomaterials can be described in terms of charge-transfer exciton theory, where two neighboring porphyrin molecules with different electronic characteristics form an electron-donor-acceptor charge-transfer complex (Segawa et al., 1989, 1992b; Knoester and Agranovich, 2003; Scholes and Rumbles, 2006; Zhu et al., 2009; Martin et al., 2010; Natali and Scandola, 2016). This theory assumes that the charge-transfer excitons in the electron-donor-acceptor complexes are generated by absorption of light, and that these are essential for the creation of free carriers (Knoester and Agranovich, 2003). The locations of the hole and electron on different porphyrin molecules due to their different electron donating and electron accepting properties (Knoester and Agranovich, 2003; Martin et al., 2010; Natali and Scandola, 2016) increases the electron and hole separation distance and the probability of a free charge-carrier formation. An applied electric field may favor the charge separation (Scholes and Rumbles, 2006) and result in a photocurrent. Exciton theory has been applied to biomolecular aggregates in the light-harvesting systems of plants and several types of green bacteria, which absorb sunlight and transport the excitation energy to the reaction centers (Knoester and Agranovich, 2003).

As it was mentioned in the introduction section, Martin et al. (2010) described the photoconductance of the microscale clover-shaped assemblies of two metalloporphyrins Zn(II)TPPS^4−^ and Sn(IV)T(N-EtOH-4-Py)P^4+^ in terms of the charge-transfer excitons produced in the photoexcited nanostrucutres. ZnPs were considered donors and Sn(IV)Ps were considered acceptors because of the redox potentials estimations based on the literature data for the Zn and Sn(IV)OEPs. The segregated stacking of molecules similar to that in a classical donor-acceptor organic solid TTF-TCNQ was supposed. In this configuration, the excitation with light resulted in electrons on acceptor porphyrin in columns of positive charges of the pyridinium groups, and the holes remained on porphyrin with a donor character with channels formed by the negative charges of the sulfonate groups.

The donor and acceptor character of the nanorod tectons can be estimated from the energy levels of each component. This can be approached using cyclic voltammetry, which reveals the first oxidation and reduction potentials and, as a result, the relative location of the porphyrin energy levels (Mairanovsky, 1987; Bouvet and Simon, 1990; Rieger, 1994; Kadish and Van Caemelbecke, 2003; Martin et al., 2010). Under defined conditions (Delahay, 1954; Rieger, 1994), the half-wave potentials of the compounds can be taken as an approximation to the standard potentials and it is expected that their values correlate with the electron affinity of the compounds. The electron affinity is expected to be related to the energy of the lowest unoccupied molecular orbital. We carried out cyclic voltammetry of TPPS_4_ and Sn(IV)TPyP^4+^, (Figure 7 and Table 2). Measurements were performed at pH = 2.0 and 3.5 for SnTPyP^4+^, and at pH = 4.0 for H_4_${\text{TPPS}}_{4}^{2-}$ (dimerization, self-assembly) and pH = 6.9 for H_2_${\text{TPPS}}_{4}^{4-}$, respectively. On the one hand, the measurements at higher pH were impeded by a poor solubility of Sn(IV)TPyP^4+^. On the other hand, H_4_${\text{TPPS}}_{4}^{2-}$ forms dimers, J-aggregates, and nanotubes in neutral and acidic media (pH < 4.8), respectively, and the redox peaks were very sluggish and poorly defined. In general, adsorption also complicates the electrochemical measurements of TPPS_4_ and Sn(IV)TPyP compounds. Therefore, we used a negative shift of 0.030 V pH^−1^ for the first reduction of SnTPyP (found from the values taken at pH 2.0 and 3.5) to estimate ${\text{E}}_{1/2}^{\text{red}}$ of about −0.534 V for SnTPyP at pH 6.9. It is lower in energy than that found for H_2_${\text{TPPS}}_{4}^{4-}$ at this pH. The oxidation peak could be resolved only for H_2_${\text{TTPS}}_{4}^{4-}$ at pH 6.9. The oxidation peak of Sn(IV)TPyP is at more positive potentials interfering with the decomposition of the aqueous solutions. The CV of the NS adsorbed overnight on a GCE (washed thoroughly with water before measurements) revealed a broad feature corresponding presumably to the reduction of the Sn(IV)TPyP species at a lower potential window of about −0.6 to −0.4 V (Ag/AgCl). It also revealed a reduction process at a higher energy of about −0.7 V (Ag/AgCl), (Figure 7), presumably corresponding to the reduction processes of the TPPS_4_ species. However, it was shown that the redox potentials of the porphyrins may be shifted due to their interactions, e.g., as shown for the porphyrin ion-paired porphyrin dimers (Natali and Scandola, 2016). Altogether, the data suggest that Sn(IV)TPyP can be considered as a molecule with more acceptor properties and TPPS_4_ as a molecule with more donor properties in this couple. Indeed, this is in agreement with the fact that the Sn(IV) complex is considered one of the most electropositive metalloporphyrins (Fuhrhop et al., 1973; Koposova et al., 2016a). While it is stable against electrophilic attack, it is very reactive with reducing agents (Fuhrhop et al., 1973). Moreover, the Py substituents of the porphyrin ring have more electron-withdrawing properties than 4-sulfonatophenyl substituents, contributing to lowering the reduction potential of Sn(IV) porphyrins with Py substituents of the macrocycle and the stability of a π-radical anion of Sn(IV)P (Jahan et al., 2012; Koposova et al., 2016a). It is worth mentioning that the CV of the adsorbed nanostructures indicates that the energy levels of individual molecules may be changed in the nanostrucutres, as shown for the porphyrin ion pairs (Natali and Scandola, 2016).

**Table 2:** Half-wave reduction (${\text{E}}_{1/2}^{\text{red}}$) and oxidation ${\text{E}}_{\text{p}}^{\text{ox}}$ potentials of TPPS_4_ and Sn(IV)TPyP^4+^ at different pH in aqueous solution.

**Porphyrin**	**E**_****1/2****_^****red****^**, ${\text{E}}_{\text{p}}^{\text{ox}}$, V**			
	**pH = 2.0**	**pH = 3.5**	**pH = 4.0**	**pH = 6.9**
TPPS_4_			(−0.434)^a^	−0.703
			–	+0.868
Sn(IV)TPyP	−0.384	−0.429	–	(−0.534)^b^
	>1.40	>1.40		–

a *Possible formation of dimers or larger aggregates*.

b *Calculated assuming ca. 30 mV pH^−1^ for Sn(IV)TPyP*.

Thus, applying this principle to the couple under investigation and based on the cyclic voltammetry data we can assume that photoirradiation may lead to exciton delocalization in the H_4_${\text{TPPS}}_{4}^{2-}$ –Sn(IV)TPyP^4+^ porphyrin nanotubes, where Sn(IV)TPyP porphyrin possesses an acceptor character and H_4_${\text{TPPS}}_{4}^{2-}$ porphyrin possesses a donor character in this couple, although, the positions of LUMO and HOMO for Sn(IV)TPyP^4+^ and H_4_${\text{TPPS}}_{4}^{2-}$ are difficult to define at the same conditions. However, donor-acceptor interactions and charge-transfer exciton may appear not only in systems with two types of molecules but for one type of molecules such as chlorophyll (Katz, 1979), in the case of separation and transportation of the photogenerated electron-hole pairs in the 5,10,15,20-tetraphenylporphyrin nanospheres (Zhang et al., 2015), or molecule crystals such as anthracene, naphthalene, etc. (Knoester and Agranovich, 2003). In the latter case, any molecule in the crystal can play the role of donor or acceptor. Additionally, it was supposed in the above-mentioned works (Franco et al., 2010; Martin et al., 2010) that the location of the hole on the positively charged porphyrins (Sn(IV)TPyP^4+^) and the electrons on the negatively charged porphyrins (H_4_${\text{TPPS}}_{4}^{2-}$) might be energetically unfavorable and result in electron-hole recombination not favoring the conductivity (Martin et al., 2010). These data also support the charge-transfer mechanism in the H_4_${\text{TPPS}}_{4}^{2-}$-Sn(IV)TPyP^4+^ system with Sn(IV)TPyP^4+^ as an electron acceptor and H_4_${\text{TPPS}}_{4}^{2-}$ as a donor.

It is interesting to compare the apparent photoconductivity of the H_4_${\text{TPPS}}_{4}^{2-}$-Sn(IV)TPyP^4+^ nanotubes with that of the H_4_${\text{TPPS}}_{4}^{2-}$-Co(III)T(4-Py)P self-assembled nanotubes (Koposova et al., 2018) and Sn(IV)TPPS_4_-Co(III)T(4-Py)P nanostructures (Koposova et al., 2016b), which we studied recently. In the first two systems, the optical UV-visible spectra of the self-assembled nanostructures exhibit J-aggregate absorbance bands at about 500 nm, 716 nm and 494 nm, 709 nm, respectively, while the absorption spectrum of the Sn(IV)TPPS_4_ - Co(III)T(4-Py)P nanostructures lacks these bands, probably because of the longer intermolecular distances, weaker intermolecular interactions, and a weaker electronic coupling in the latter system. As a result, the apparent conductivities of the H_4_${\text{TPPS}}_{4}^{2-}$-Sn(IV)TPyP^4+^ and H_4_${\text{TPPS}}_{4}^{2-}$-Co(III)T(4-Py)P self-assembled nanotubes are higher due to a higher probability of the exciton delocalization. Additional factors, which may contribute to the higher photoconductivity of the H_4_${\text{TPPS}}_{4}^{2-}$-Sn(IV)TPyP^4+^ system is closeness of the energies of unoccupied molecular orbitals and electron affinity of the porphyrins in this system as follows from the above mentioned experiments so that no completed redox processes between two porphyrins takes place, which would stop a directed electron flow. Further studies on elucidation of exact relative arrangement and packing of different porphyrin molecules in the nanostructures may be useful to explain influence of the structural features on photoconductivity in the porphyrin nanotubes.

**Figure 8:**
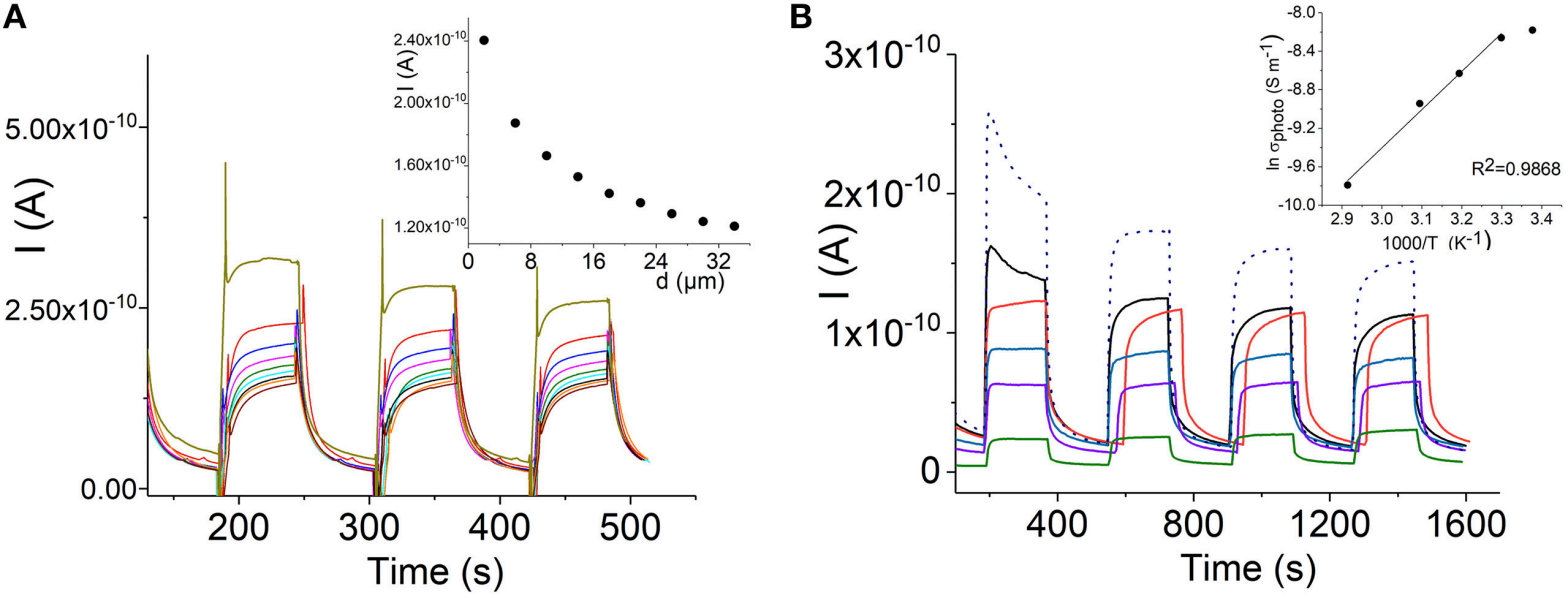
Dependence of the photocurrent of the H_4_${\text{TPPS}}_{4}^{2-}$-Sn(IV)TPyP^4+^ porphyrin nanotubes on the path length, interdigitated electrode array (see experimental section), V_app_ = 0.5 V **(A)**. Temperature dependence of the photocurrent of the H_4_${\text{TPPS}}_{4}^{2-}$ - Sn(IV)TPyP^4+^ nanotubes: 23°C–black line, 30°C– red line, 40°C–blue line, 50°C– violet line,70°C– green line, after cooling down to 23°C–dotted line. Insert shows a dependence of lnσ_photo_ on 1,000/*T*, 400 nm electrode gap, V_app_ = 0.5 V **(B)**.

Porphyrin and porphyrin nanostructures are important compounds for the development of sensors (Malinski, 2000; Guo et al., 2014; Paolesse et al., 2017; Skripnikova et al., 2017). In particular, Sn(IV)Ps are responsible for the selectivity to salicylate anions in ion-selective electrodes due to axial salicylate ligand binding to the metal center (Malinski, 2000; Skripnikova et al., 2017). In this study, we also examined the sensing properties of the H_4_${\text{TPPS}}_{4}^{2-}$ –Sn(IV)TPyP^4+^ nanorods based on their photoconductivity in a chemiresistor sensor mode (Muratova et al., 2016, Figure 8A). Figure 8B illustrates the change in the photocurrent of the nanostructures after exposure to Sal^−^-ions at room temperature. All samples were dried before the measurements. Insert in Figure 8B demonstrates a corresponding dependence of the photoconductivity of the H_4_${\text{TPPS}}_{4}^{2-}$-Sn(IV)TPyP^4+^ nanorod chemiresistor on the concentration of salicylate ions. We suppose that a relatively large error of measurements is due to a poor reproducibility of the interface between porphyrin nanorods and the contact metal electrodes, (Figure 8A). However, these experiments show a potential utility of the self-assembled porphyrin nanorods as functional layers for the sensor devices.

**Figure 9:**
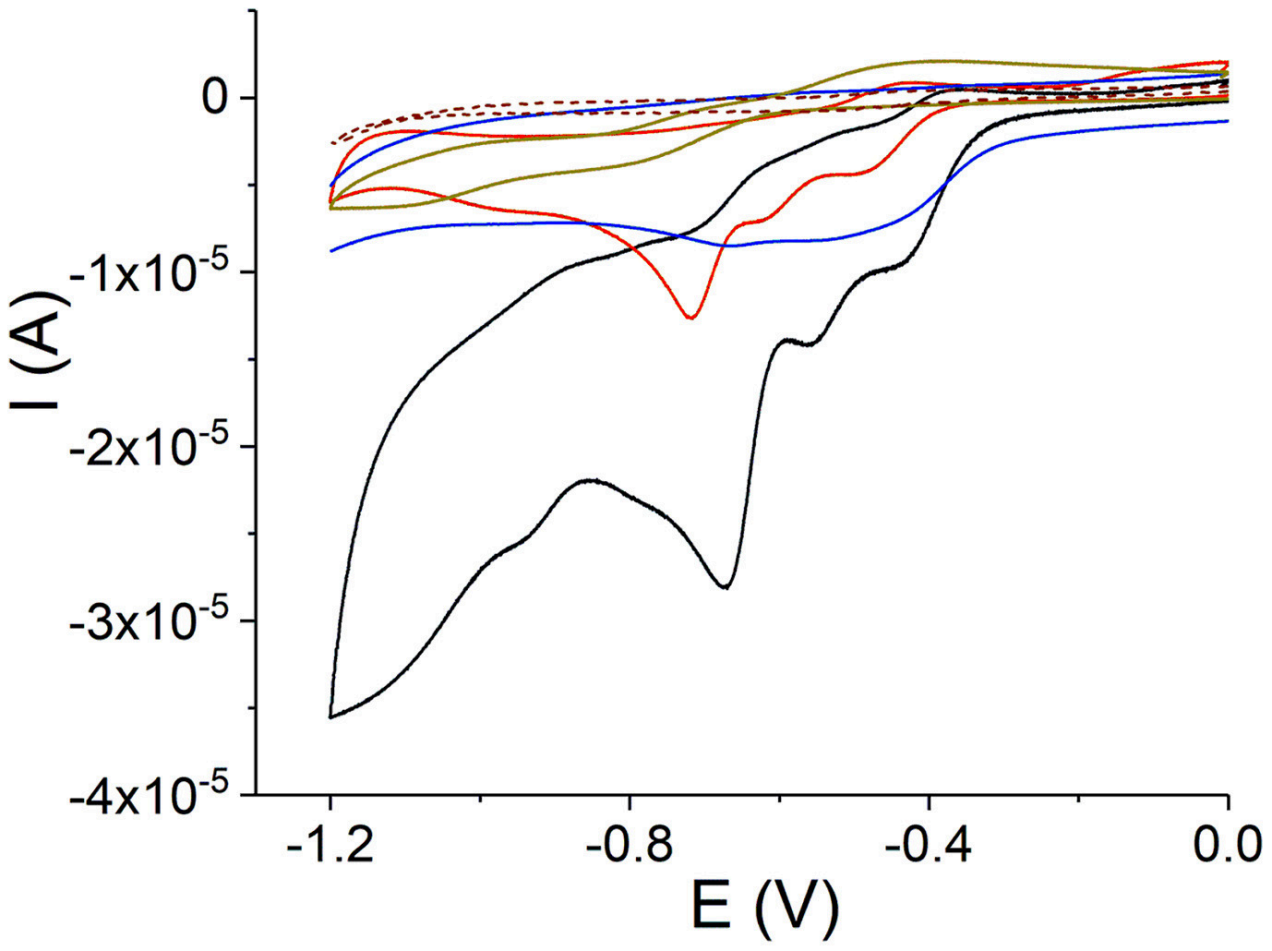
Cyclic voltammograms on a GCE: Sn(IV)TPyP^4+^ at pH 2 (black line) and 3.5 (red line), H_2_${\text{TPPS}}_{4}^{4-}$ at pH 6.9 (dark yellow line), H_4_${\text{TPPS}}_{4}^{2-}$ and aggregates at pH 4 (brown dashed line), and H_4_${\text{TPPS}}_{4}^{2-}$-Sn(IV)TPyP^4+^ adsorbed overnight (blue line), 0.1 M KNO_3_, scan rate 50 mV s^−1^.

**Figure 10:**
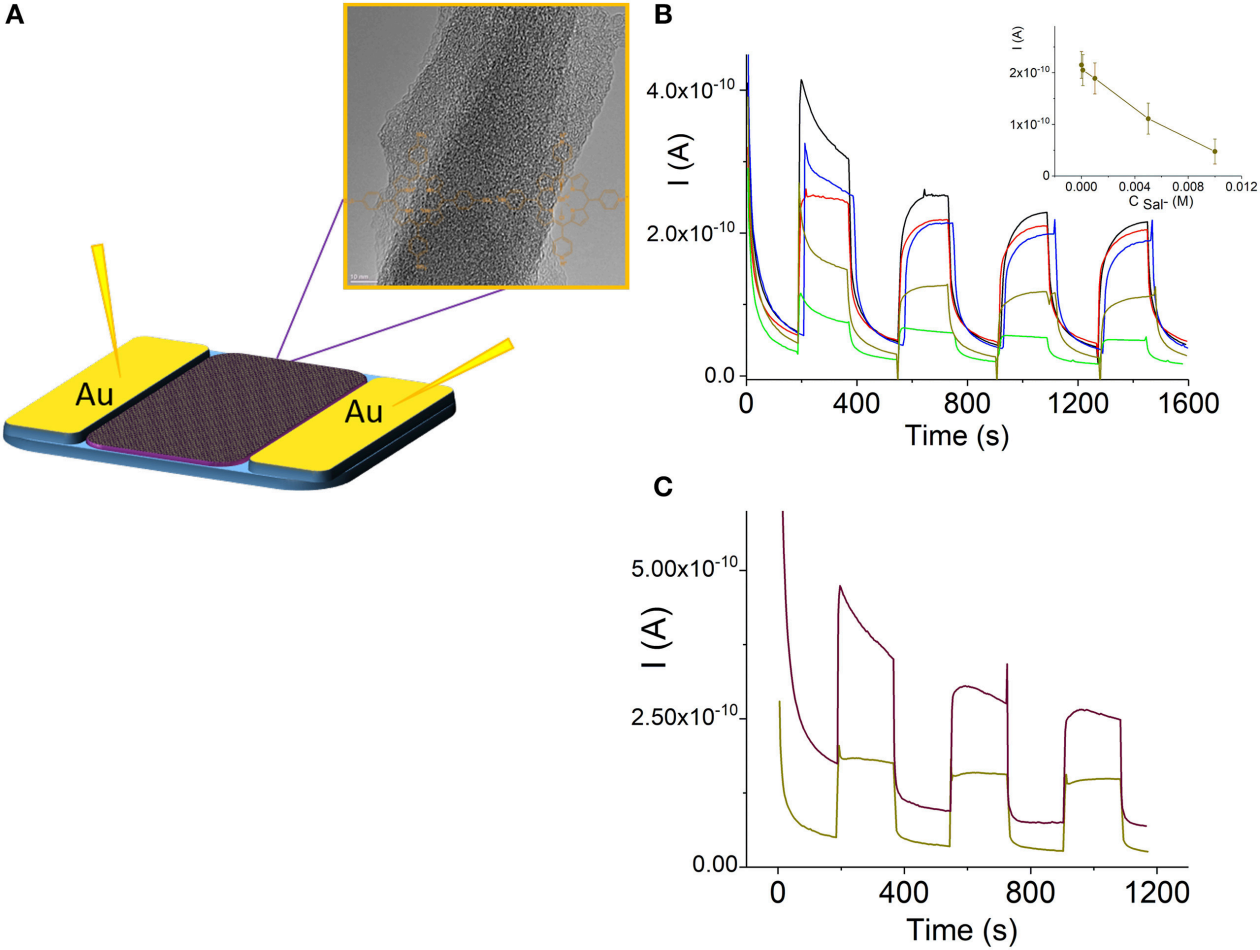
Schematic illustration of a porphyrin nanorod chemiresistor **(A)**. (Photo)current response curves of the H_4_${\text{TPPS}}_{4}^{2-}$-Sn(IV)TPyP^4+^ porphyrin nanorods in a chemoresistor sensor mode before (black line) and after exposure to salicylate 10^−4^ M (red line), 10^−3^ M (blue line), 5·10^−2^ (dark yellow line), and 10^−2^ M (green line) **(B)**, insert in **(B)** shows a corresponding dependence of the photocurrent of the H_4_${\text{TPPS}}_{4}^{2-}$-Sn(IV)TPyP^4+^ nanorod chemiresistor on the concentration of salicylate ion, *n* = 3, *p* = 0.95. (Photo)current of the H_4_${\text{TPPS}}_{4}^{2-}$-SnTPyP^4+^ porphyrin nanorods before (dark yellow line) and after (brown line) exposure to iodine vapor for 60 min, V_app_ = 0.5 V **(C)**.

Both the dark current and photoconductance of the H_4_${\text{TPPS}}_{4}^{2-}$-SnTPyP^4+^ nanorods increased in case of exposure to iodine vapor, Figure 10C. The photocurrents of porphyrin films pretreated with iodine or oxygen electron acceptors were found to be higher than those without pretreatment (Yamashita and Maenobe, 1980; Hoffman and Ibers, 1983; Zhang et al., 1995; Savenije and Goossens, 2001). The contribution of acceptor impurities, e.g., O_2_ or iodine, should be taken into account in the exciton mechanism of the photoconductance of porphyrins, where a charge-transfer complex formation with an acceptor such as iodine (Hoffman and Ibers, 1983) or oxygen (Kobayashi et al., 1993) was proposed. Recently, a nanoelectronic chemosensor was proposed for the detection of vapor-phase H_2_O_2_ based on the self-assembled Ti porphyrin (Guo et al., 2014). It can be assumed that hydrogen peroxide influences the number of charge carriers in the porphyrin nanostructure-based channel, which would be responsible for the sensor sensitivity.

The absorption spectrum of SnTPyP^4+^-H_4_${\text{TPPS}}_{4}^{2-}$ porphyrin nanotubes has a series of absorption bands in 400–750 nm region, (Figure 4). Previous studies have shown that the photoconductivity is observed at a laser illumination of 488 nm (close to the absorption wavelength of J-aggregates) in the TPPS_4_ and TPPS_3_ nanorods (Schwab et al., 2004; Yeats et al., 2008), any wavelength of absorption in TTP (Weigl, 1957), and at different wavelengths with different intensity according to the absorption spectra of the nanorods assembled from TSPP and TMPyP or TPyP (Adinehnia et al., 2016; Borders et al., 2017). In this study, we found that the Q-band region with a Q-band of Sn(IV)TPyP^4+^ (550–552 nm) and the J-band of nanostructures at 714 nm are responsible for about 34% of photoconductance of the self-assembled H_4_${\text{TPPS}}_{4}^{2-}$-Sn(IV)TPyP^4+^ porphyrin nanotubes by exposure to visible light irradiation with an additional 550 nm longpass filter.

## Conclusions

We investigated morphological, spectral, and electrical properties of the self-assembled H_4_${\text{TPPS}}_{4}^{2-}$-Sn(IV)TPyP^4+^ tubular nanostructures formed by monomers at pH = 2.0. The formation of hollow tubes can include a series of mechanisms of self-assembly assuming a combination of a series of intermolecular interactions. The H_4_${\text{TPPS}}_{4}^{2-}$ –Sn(IV)TPyP^4+^ nanotube network demonstrates a photoconductivity under visible light in a semiconductor range of (3.1 ± 0.9) × 10^−4^ S m^−1^. The temperature dependence of the photoconductance of the nanotubes showed a metal-like character of decreasing current with increasing temperature. The photocurrent decreased over the μm distances. It was found that excitation of the Q-band region with a Q-band of Sn(IV)TPyP^4+^ (550–552 nm) and the J-band of the nanostructures at 714 nm is responsible for about 34% of the photoconductance activity of the H_4_${\text{TPPS}}_{4}^{2-}$ –Sn(IV)TPyP^4+^- porphyrin nanotubes. The sensor properties of the nanotubes in a chemiresistor mode were tested to demonstrate a perspective of the self-assembled porphyrin nanorods as functional layers for the sensor devices and biomimetic nanoarchitectures.

## Data Availability

All datasets generated for this study are included in the manuscript and/or the Supplementary Files.

## Author Contributions

EK made substantial contributions to design, acquisition, analysis, and interpretation of data, and participated in drafting the article. AO and YE made substantial contributions to conception, analysis, and interpretation of data, and participated in drafting the article. YM made substantial contributions to conception, design, acquisition, analysis, and interpretation of data, and participated in drafting the article. All authors gave final approval of the submitted manuscript.

### Conflict of Interest Statement

The authors declare that the research was conducted in the absence of any commercial or financial relationships that could be construed as a potential conflict of interest.

## References

[B1] Adinehnia M. Borders B. Ruf M. Chilukuri B. Hipps K. W. Mazur U. (2016). Comprehensive structure–function correlation of photoactive ionic π-conjugated supermolecular assemblies: an experimental and computational study. J. Mater. Chem. C 4, 10223–10239. 10.1039/C6TC03957J

[B2] Agranovich E. V. M. Bassani G. F. (2003). Electronic Excitations in Organic Based Nanostructures. Oxford, UK: Elsevier Academic Press.

[B3] Borders B. Adinehnia M. Chilukuri B. Ruf M. Hipps K. W. Mazur U. (2018). Tuning the optoelectronic characteristics of ionic organic crystalline assemblies. J. Mater. Chem. C 6, 4041–4056. 10.1039/C8TC00416A

[B4] Borders B. Adinehnia M. Rosenkrantz N. Van Zijll M. Hipps K. W. Mazur U. (2017). Photoconductive behavior of binary porphyrin crystalline assemblies. J. Porphyrins Phthalocyan. 21, 569–580. 10.1142/S1088424617500638

[B5] Bouvet M. Simon J. (1990). Electrical properties of rare earth bisphthalocyanine and bisnaphthalocyanine complexes. Chem. Phys. Lett. 172, 299–302. 10.1016/0009-2614(90)85407-4

[B6] Cai J. Chen H. Huang J. Wang J. Tian D. Dong H.. (2014). Controlled self-assembly and photovoltaic characteristics of porphyrin derivatives on a silicon surface at solid-liquid interfaces. Soft Matter 10, 2612–2618. 10.1039/c3sm53061b 24647426

[B7] Chen Y. Li A. Huang Z.-H. Wang L.-N. Kang F. (2016). Porphyrin-based nanostructures for photocatalytic applications. Nanomaterials 6:51. 10.3390/nano6030051 28344308 PMC5302509

[B8] Chou J. H. Kosal M. E. Nalwa H. S. Rakow N. A. Suslick K. S. (2000). Application of porphyrins and metalloporphyrins of materials chemistry, in The Porphyrin Handbook, eds Kadish K. M. Smith K. M. Guilard R. (San Diego, CA: Academic Press), 346.

[B9] Collman J. P. Kendall J. L. Chen J. L. Collins K. A. Marchon J.-C. (2000). Formation of charge-transfer complexes from neutral bis(porphyrin) sandwiches. Inorg. Chem. 39, 1661–1667. 10.1021/ic9907516 12526551

[B10] Delahay P. (1954). New Instrumental Methods in Electrochemistry. New York, NY: Interscience Publ. Inc.

[B11] Drain C. M. (2002). Self-organization of self-assembled photonic materials into functional devices: photo-switched conductors. Proc. Natl. Acad. Sci. U.S.A. 99, 5178–5182. 10.1073/pnas.062635099 11943850 PMC122742

[B12] El-Khouly M. E. Fukuzumi S. D'souza F. (2014). Photosynthetic antenna–reaction center mimicry by using boron dipyrromethene sensitizers. ChemPhysChem 15, 30–47. 10.1002/cphc.201300715 24243758

[B13] Ferraris J. Walatka V. Perlstein J. Cowan D. O. (1973). Electron-transfer in a new highly conducting donor-acceptor complex. J. Am. Chem. Soc. 95, 948–949. 10.1021/ja00784a066

[B14] Franco R. Jacobsen J. L. Wang H. Wang Z. Istvan K. Schore N. E.. (2010). Molecular organization in self-assembled binary porphyrin nanotubes revealed by resonance raman spectroscopy. Phys. Chem. Chem. Phys. 12, 4072–4077. 10.1039/b926068d 20379498

[B15] Friesen B. A. Wiggins B. Mchale J. L. Mazur U. Hipps K. W. (2010). Differing HOMO and LUMO mediated conduction in a porphyrin nanorod. J. Am. Chem. Soc. 132, 8554–8556. 10.1021/ja103078q 20524617

[B16] Fuhrhop J. H. (2014). Porphyrin assemblies and their scaffolds. Langmuir 30, 1–12. 10.1021/la402228g 24138176

[B17] Fuhrhop J. H. Kadish K. M. Davis D. G. (1973). The redox behavior of metallooctaethylporphyrins. J. Am. Chem. Soc. 95, 5140–5147. 10.1021/ja00797a008 4354829

[B18] Fukuzumi S. Imahori H. (2008). Biomimetic electron-transfer chemistry of porphyrins and metalloporphyrins, in Electron Transfer in Chemistry, ed V. Balzani (Weinheim: Wiley-VCH Verlag GmbHGmbH), 927–975. 10.1002/9783527618248.ch31

[B19] George R. C. Egharevba G. O. Nyokong T. (2010). Spectroscopic studies of nanostructures of negatively charged free base porphyrin and positively charged tin porphyrins. Polyhedron 29, 1469–1474. 10.1016/j.poly.2010.01.028

[B20] Golubchikov O. A. Berezin B. D. (1986). Applied aspects of the chemistry of porphyrins. Uspehi Chimii 8, 1361–1389. 10.1070/RC1986v055n08ABEH003221

[B21] Guldi D. M. Imahori H. (2004). Supramolecular assemblies for electron transfer. J. Porphyrins Phthalocyanines 8, 976–983. 10.1142/S1088424604000337

[B22] Guo P. Zhao G. Chen P. Lei B. Jiang L. Zhang H. (2014). Porphyrin nanoassemblies via surfactant-assisted assembly and single nanofiber nanoelectronic sensors for high-performance H_2_O_2_ vapor sensing. ACS Nano 8, 3402–3411. 10.1021/nn406071f 24654963

[B23] Hoffman B. M. Ibers J. A. (1983). Porphyrinic molecular metals. Acc. Chem. Res. 16, 15–21. 10.1021/ar00085a003

[B24] Jahan M. Bao Q. Loh K. P. (2012). Electrocatalytically active graphene–porphyrin MOF composite for oxygen reduction reaction. J. Am. Chem. Soc. 134, 6707–6713. 10.1021/ja211433h 22439970

[B25] Jana A. Ishida M. Park J. S. Bähring S. Jeppesen J. O. Sessler J. L. (2017). Tetrathiafulvalene- (TTF-) derived oligopyrrolic macrocycles. Chem. Rev. 117, 2641–2710. 10.1021/acs.chemrev.6b00375 27753290

[B26] Jiang B. Yang S.-W. Jones W. E. (1997). Conjugated porphyrin cpolymers: control of chromophore separation by oligophenylenevinylene bridges. Chem. Mat. 9, 2031–2034. 10.1021/cm970225h

[B27] Jones R. Tredgold R. H. Hodge P. (1983). Langmuir-Blodgett films of simple esterified porphyrins. Thin Solid Films 99, 25–32. 10.1016/0040-6090(83)90355-3

[B28] Kadish K. M. Van Caemelbecke E. (2003). Electrochemistry of porphyrins and related macrocycles. J. Solid State Electrochem. 7, 254–258. 10.1007/s10008-002-0306-3

[B29] Katz J. J. (1979). Charge separation in synthetic photoreaction centers, in Light-Induced Charge Separation in Biology and Chemistry, eds Gerischer H. Katz J. J. (Weinheim: Chemie), 331–359.

[B30] Knoester J. Agranovich V. M. (2003). Frenkel and charge-transfer excitons in organic solids, in Electronic Excitations in Organic Based Nanostructures, eds Agranovich V. M. Bassani G. F. (Oxford, UK: Elsevier Academic Press), 508 10.1016/S1079-4050(03)31001-4

[B31] Kobayashi N. Andrew Nevin W. Mizunuma S. Awaji H. Yamaguchi M. (1993). Ring-expanded porphyrins as an approach towards highly conductive molecular semiconductors. Chem. Phys. Lett. 205, 51–54. 10.1016/0009-2614(93)85165-K

[B32] Kocherzhenko A. A. Patwardhan S. Grozema F. C. Anderson H. L. Siebbeles L. D. A. (2009). Mechanism of charge transport along zinc porphyrin-based molecular wires. J. Am. Chem. Soc. 131, 5522–5529. 10.1021/ja809174y 19331354

[B33] Koposova E. Liu X. Pendin A. Thiele B. Shumilova G. Ermolenko Y. (2016a). Influence of meso-substitution of the porphyrin ring on enhanced hydrogen evolution in a photochemical system. J. Phys. Chem. C 120, 13873–13890. 10.1021/acs.jpcc.6b01467

[B34] Koposova E. A. Ermolenko Y. E. Offenhäusser A. Mourzina Y. G. (2018). Self-assembly and photoconductivity of binary porphyrin nanostructures of meso-tetrakis(4-sulfonatophenyl)porphine and Co(III) meso-tetra(4-pyridyl)porphine chloride. Colloid Surf. A Physicochem. Eng. Asp. 548, 172–178. 10.1016/j.colsurfa.2018.03.053

[B35] Koposova E. A. Pendin A. A. Ermolenko Y. E. Shumilova G. I. Starikova A. A. Mourzina Y. G. (2016b). Morphological properties and photoconductivity of self-assembled Sn/Co porphyrin nnostructures. Rev. Adv. Mater. Sci. 45, 15–19. Available online at: http://www.ipme.ru/e-journals/RAMS/no_14516/03_14516_koposova.pdf

[B36] Li G. Wang T. Schulz A. Bhosale S. Lauer M. Espindola P. (2004). Porphyrin-acetylene-thiophene polymer wires. Chem. Commun. 2004, 552–553. 10.1039/B313415F 14973603

[B37] Mairanovsky V. G. (1987). Electrochemistry of porphyrins, in Porphyrins: Spectroscopy, Electrochemistry, Application, ed N. Enikolopyan (Moscow: Nauka), 127–181.

[B38] Maiti N. C. Ravikanth M. Mazumdar S. Periasamy N. (1995). Fluorescence dynamics of non-covalently linked porphyrin dimers and aggregates. J. Phys. Chem. 99, 17192–17197. 10.1021/j100047a024

[B39] Malinski T. (2000). Porphyrin-based electrochemical sensors, in The Porphyrin Handbook, eds Kadish K. M. Smith K. M. Guilard R. (San Diego, CA: Academic Press), 232–256.

[B40] Martin K. E. Tian Y. Busani T. Medforth C. J. Franco R. Van Swol F. (2013). Charge effects on the structure and composition of porphyrin binary ionic solids: ZnTPPS/SnTMePyP nanomaterials. Chem. Mat. 25, 441–447. 10.1021/cm303595s

[B41] Martin K. E. Wang Z. Busani T. Garcia R. M. Chen Z. Jiang Y. (2010). Donor–acceptor biomorphs from the ionic self-assembly of porphyrins. J. Am. Chem. Soc. 132, 8194–8201. 10.1021/ja102194x 20469866

[B42] Mchale J. L. (2012). Hierarchal structure of light-harvesting porphyrin aggregates, in J-Aggregates, ed T. Kobayashi (Singapore: World Scientific), 77–118. 10.1142/9789814365796_0003

[B43] Medforth C. J. Wang Z. Martin K. E. Song Y. Jacobsen J. L. Shelnutt J. A. (2009). Self-assembled porphyrin nanostructures. Chem. Commun. 2009, 7261–7277. 10.1039/b914432c 20024202

[B44] Mirkovic T. Ostroumov E. E. Anna J. M. Van Grondelle R. Govindjee Scholes G. D. (2017). Light absorption and energy transfer in the antenna complexes of photosynthetic organisms. Chem. Rev. 117, 249–293. 10.1021/acs.chemrev.6b00002 27428615

[B45] Muratova I. S. Mikhelson K. N. Ermolenko Y. E. Offenhäusser A. Mourzina Y. (2016). Chemiresistors based on ultrathin gold nanowires for sensing halides, pyridine and dopamine. Sens. Actuator B Chem. 232, 420–427. 10.1016/j.snb.2016.03.151

[B46] Naarman H. (2012). Polymers, electrically conducting, in Ullmann's Encyclopedia of Industrial Chemistry, ed B. Elvers (Weinheim: Wiley-VCH Verlag GmbH), 295–314.

[B47] Natali M. Scandola F. (2016). Photoinduced charge separation in porphyrin ion pairs. J. Phys. Chem. A 120, 1588–1600. 10.1021/acs.jpca.6b00960 26905260

[B48] Ohno O. Kaizu Y. Kobayashi H. (1993). J-aggregate formation of a water-soluble porphyrin in acidic aqueous media. J. Chem. Phys. 99, 4128–4139. 10.1063/1.466109

[B49] Ou X. Chen P. Jiang L. Shen Y. Hu W. Liu M. (2014). π-Conjugated molecules crosslinked graphene-based ultrathin films and their tunable performances in organic nanoelectronics. Adv. Funct. Mater. 24, 543–554. 10.1002/adfm.201302153

[B50] Paolesse R. Nardis S. Monti D. Stefanelli M. Di Natale C. (2017). Porphyrinoids for chemical sensor applications. Chem. Rev. 117, 2517–2583. 10.1021/acs.chemrev.6b00361 28222604

[B51] Pasternack R. F. Huber P. R. Boyd P. Engasser G. Francesconi L. Gibbs E.. (1972). Aggregation of meso-substituted water-soluble porphyrins. J. Am. Chem. Soc. 94, 4511–4517. 10.1021/ja00768a016 5036163

[B52] Rieger P. H. (1994). Electrochemistry. New York, NY: Chapman & Hall Inc 10.1007/978-94-011-0691-7

[B53] Riley C. K. Muller E. A. Feldman B. E. Cross C. M. Aken K. L. V. Johnston D. E. (2010). Effects of O_2_, Xe, and gating on the photoconductivity and persistent photoconductivity of porphyrin nanorods. J. Phys. Chem. C 114, 19227–19233. 10.1021/jp1068494

[B54] Rosaria L. D'urso A. Mammana A. Purrello R. (2008). Chiral memory: induction, amplification, and switching in porphyrin assemblies. Chirality 20, 411–419. 10.1002/chir.20464 17806090

[B55] Savenije T. J. Goossens A. (2001). Hole transport in porphyrin thin films. Phys. Rev. B 64:115323 10.1103/PhysRevB.64.115323

[B56] Schall A. P. Iavicoli P. Qi Z. J. Menko J. Lu Y. Linares M. (2015). Photoconductivity of nanofilaments that are self-assembled from a porphyrin with long alkyl-chain substituents. J. Phys. Chem. C 119, 26154–26163. 10.1021/acs.jpcc.5b07902

[B57] Scholes G. D. Rumbles G. (2006). Excitons in nanoscale systems. Nat. Mater. 5, 683–697. 10.1038/nmat1710 16946728

[B58] Schramm C. J. Scaringe R. P. Stojakovic D. R. Hoffman B. M. Ibers J. A. Marks T. J. (1980). Chemical, spectral, structural, and charge transport properties of the molecular metals produced by iodination of nickel phthalocyanine. J. Am. Chem. Soc. 102, 6702–6713. 10.1021/ja00542a008

[B59] Schwab A. D. Smith D. E. Bond-Watts B. Johnston D. E. Hone J. Johnson A. T. (2004). Photoconductivity of self-assembled porphyrin nanorods. Nano Lett. 4, 1261–1265. 10.1021/nl049421v

[B60] Segawa H. Nakayama N. Shimidzu T. (1992a). Electrochemical synthesis of one-dimensional donor–acceptor polymers containing oligothiophenes and phosphorus porphyrins. J. Chem. Soc. Chem. Commun. 1992, 784–786. 10.1039/C39920000784

[B61] Segawa H. Nishino H. Kamikawa T. Honda K. Shimidzu T. (1989). Hetero-aggregation between gold porphyrins and zinc porphyrins through charge transfer interaction. Chem. Lett. 18, 1917–1920. 10.1246/cl.1989.1917

[B62] Segawa H. Takehara C. Honda K. Shimidzu T. Asahi T. Mataga N. (1992b). Photoinduced electron-transfer reactions of porphyrin heteroaggregates: energy gap dependence of an intradimer charge recombination process. J. Phys. Chem. 96, 503–506. 10.1021/j100181a001

[B63] Skripnikova T. A. Starikova A. A. Shumilova G. I. Ermolenko Y. E. Pendin A. A. Mourzina Y. G. (2017). Towards stabilization of the potential response of Mn(III) tetraphenylporphyrin-based solid-state electrodes with selectivity for salicylate ions. J. Solid State Electrochem. 21, 2269–2279. 10.1007/s10008-017-3575-6

[B64] Torres T. Bottari G. (2013). Organic Nanomaterials: Synthesis, Characterization, and Device Applications. Hoboken, NJ: John Wiley & Sons Inc 10.1002/9781118354377

[B65] Wang Z. Medforth C. J. Shelnutt J. A. (2004). Porphyrin nanotubes by ionic self-assembly. J. Am. Chem. Soc. 126, 15954–15955. 10.1021/ja045068j 15584716

[B66] Weigl J. W. (1957). Spectroscopic properties of organic photoconductors. Part IV. TetraphenyIporphine. J. Mol. Spectrosc. 1, 216–222. 10.1016/0022-2852(57)90024-3

[B67] Würthner F. Kaiser T. E. Saha-Möller C. R. (2011). J-Aggregates: from serendipitous discovery to supramolecular engineering of functional dye materials. Angew. Chem. Int. Edit. 50, 3376–3410. 10.1002/anie.201002307 21442690

[B68] Yamashita K. Maenobe K. (1980). Extrinsic photoconduction in metalloporphyrin films. Chem. Lett. 1980, 307–310. 10.1246/cl.1980.307

[B69] Yeats A. L. Schwab A. D. Massare B. Johnston D. E. Johnson A. T. De Paula J. C. (2008). Photoconductivity of self-assembled nanotapes made from meso-Tri(4-sulfonatophenyl)monophenylporphine. J. Phys. Chem. C 112, 2170–2176. 10.1021/jp0765695

[B70] Zhang X. Wang Y. Chen P. Guo P. Liu M. (2015). A general protocol for π-conjugated molecule-based micro/nanospheres: artificial supramolecular antenna in terms of heterogeneous photocatalysis. RSC Adv. 5, 78427–78435. 10.1039/C5RA13283E

[B71] Zhang X. Q. Wu H. M. Wu X. J. Cheng Z. P. Wei Y. (1995). Synthesis, orientation and conductivity investigation of a new porphyrin Langmuir-Blodgett film. J. Mater. Chem. 5, 401–404. 10.1039/jm9950500401

[B72] Zhu X. Y. Yang Q. Muntwiler M. (2009). Charge-transfer excitons at organic semiconductor surfaces and interfaces. Acc. Chem. Res. 42, 1779–1787. 10.1021/ar800269u 19378979

