# Supplementary material for: Photoresponsive Porphyrin Nanotubes of Meso-tetra(4-Sulfonatophenyl)Porphyrin and Sn(IV) meso-tetra(4-pyridyl)porphyrin
Source: Front Chem. 2019 May 16;7:351. doi: 10.3389/fchem.2019.00351 (PMC6532121; doi:10.3389/fchem.2019.00351)
Supplement: Supplementary file 1 [file Image_1.pdf]

Supporting information

Photoresponsive Porphyrin Nanotubes of *meso*-tetra(4-sulfonatophenyl)porphyrin and Sn(IV) *meso*-tetra(4-pyridyl)porphyrin.

Ekaterina A. Koposova[1,2], Andreas Offenhäusser[1], Yuri E. Ermolenko[2], Yulia G. Mourzina[1*]

[1] *Forschungszentrum Jülich, Institute of Complex Systems-8 (Bioelectronics), Jülich, Germany,*
[2] *Institute of Chemistry, Saint-Petersburg State University, Saint-Petersburg, Russia*

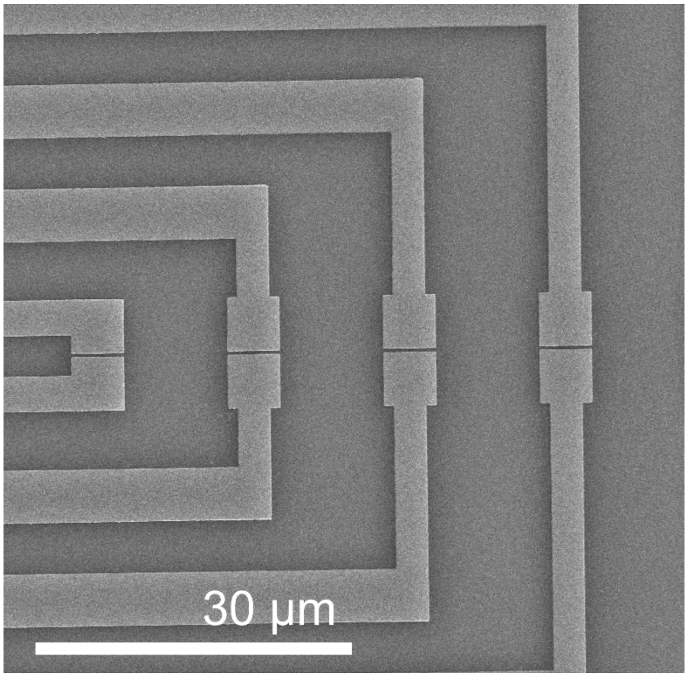

Figure S1 | Thin-film metal electrodes for the measurement of the (photo)conductance.

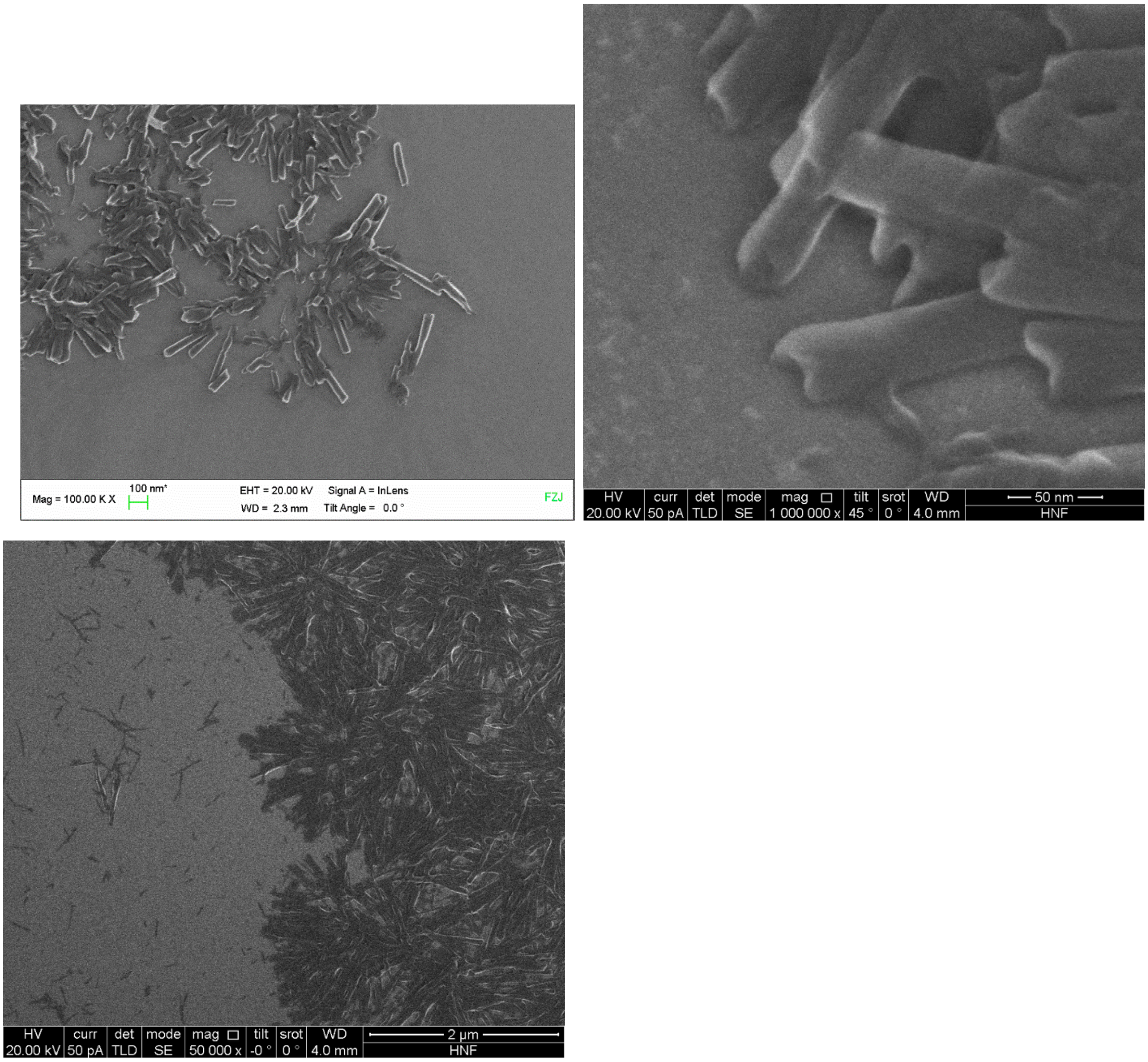

Figure S2 | SEM images of the H_4TPPS^{2-} - Sn(IV)$TPPyP^{4+}$ nanotubes formed at pH=2.0 in an equimolar solution **(A,B)** and Sn(IV)$TPyP^{4+}$ and $H_4TPPS_4^{2-}$ taken in a 1:5 concentration ratio **(C),** where the second type of nanostructures, thinner nanorods, can be observed.

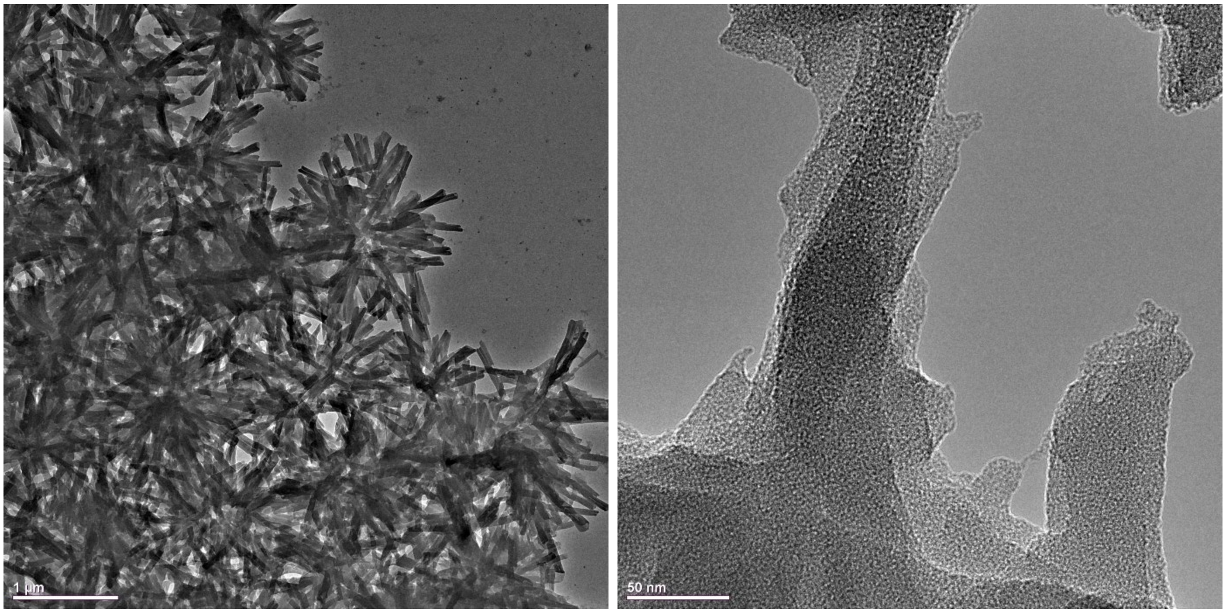

Figure S3 | TEM images of the H_4TPPS^{2-} - Sn(IV)$TPPyP^{4+}$ nanotubes formed in an equimolar solution at pH=2.0.

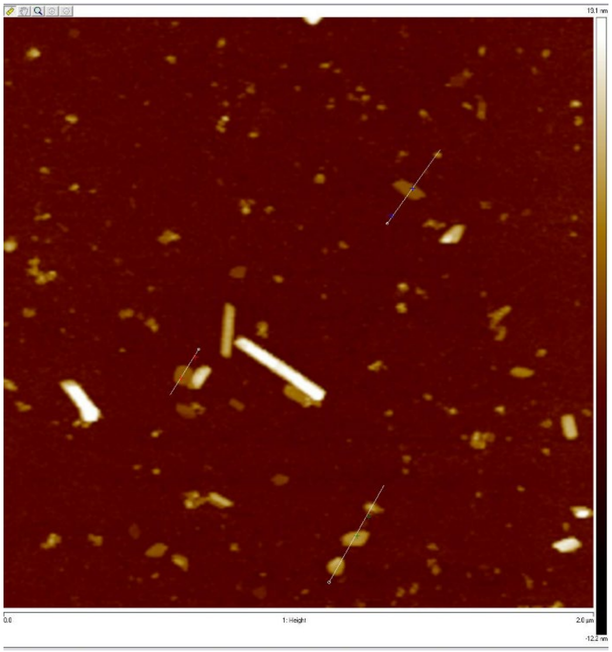

Figure S4 | AFM image of the H_4TPPS^{2-} - Sn(IV)$TPPyP^{4+}$ nanostructures formed in solutions at pH=2.0 and Sn(IV)$TPyP^{4+}$ and $H_4TPPS_4^{2-}$ taken in a 1:5 concentration ratio in solution.

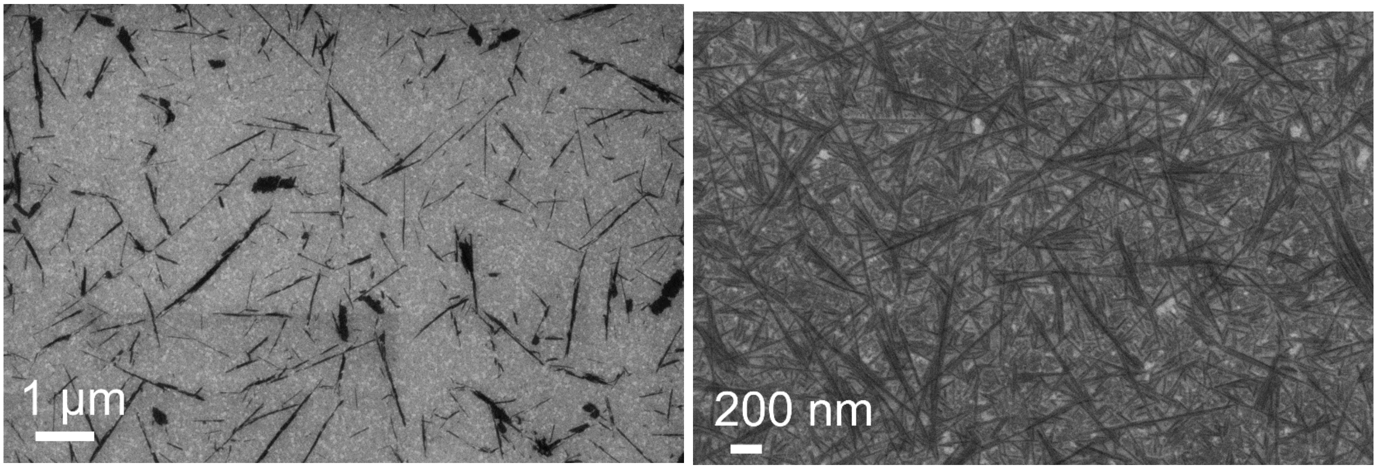

Figure S5 | SEM images of the H_4TPPS^{2-} nanotubes prepared at pH 0.94.

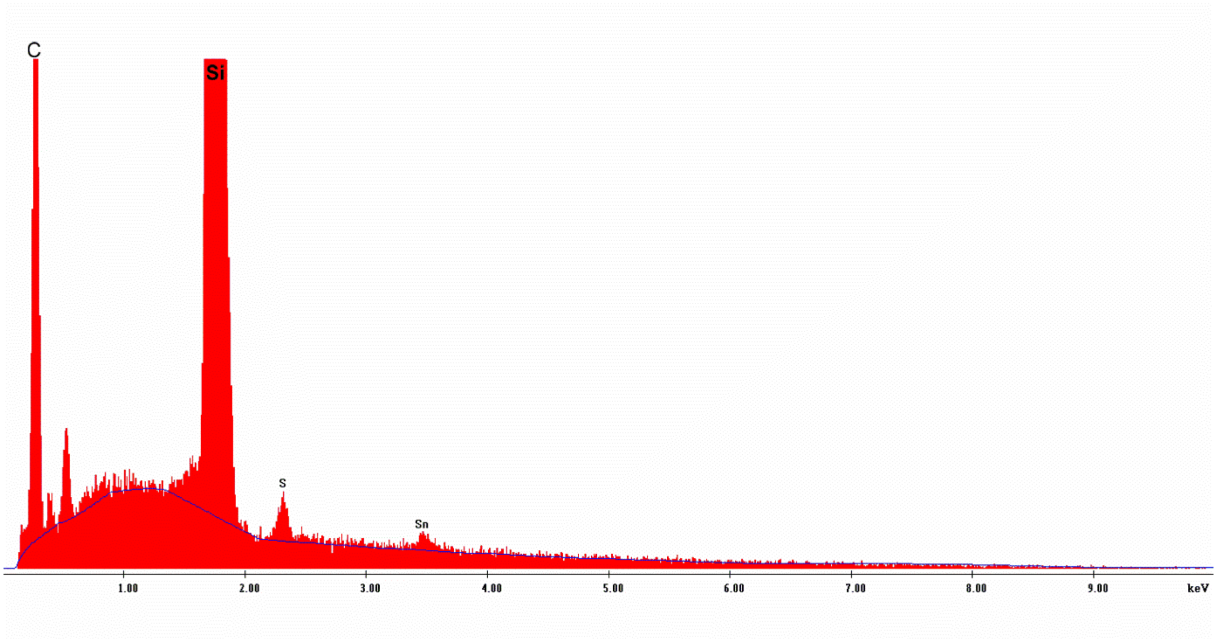

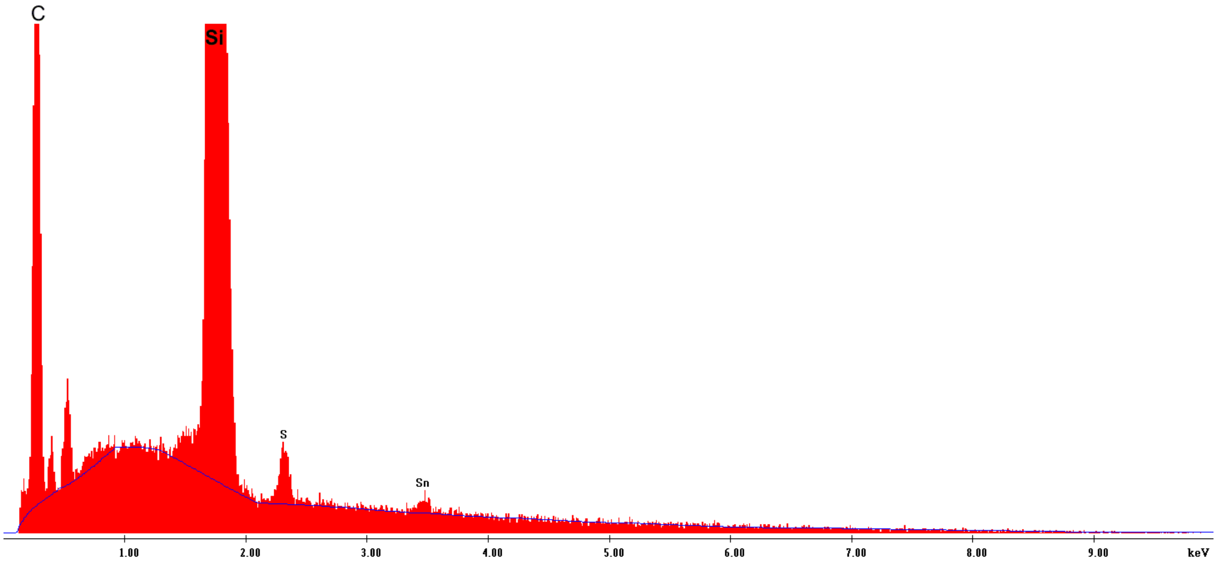

Figure S6 | EDS analyses of the H_4TPPS^{2-} - $Sn(IV)TPPyP^{4+}$ nanotubes formed in solutions at pH=2.0 and the $Sn(IV)TPyP^{4+}$ and $H_4TPPS_4^{2-}$ concentration ratio in solutions 1:5 **(A)** and 5:1 **(B)**.

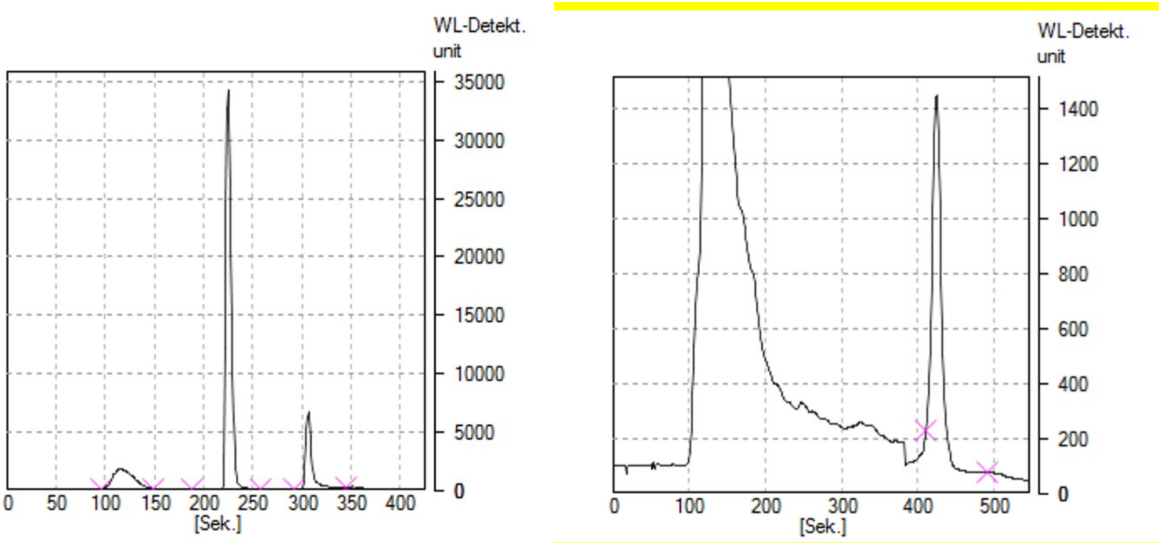

Figure S7 | Elemental analysis of the H_4TPPS^{2-} - $Sn(IV)TPPyP^{4+}$ nanotubes prepared in an equimolar solution at pH 2. Determination of CHO **(A)** and S **(B)**.
